# The transition of smooth muscle cells from a contractile to a migratory, phagocytic phenotype: direct demonstration of phenotypic modulation

**DOI:** 10.1113/JP272729

**Published:** 2016-08-13

**Authors:** Mairi E. Sandison, John Dempster, John G. McCarron

**Affiliations:** ^1^ Strathclyde Institute of Pharmacy and Biomedical SciencesUniversity of StrathclydeSIPBS Building, 161 Cathedral StreetGlasgowG4 0REUK

**Keywords:** phenotypic modulation, vascular remodeling, vascular smooth muscle

## Abstract

**Key points:**

- Smooth muscle cell (SMC) phenotypic conversion from a contractile to a migratory phenotype is proposed to underlie cardiovascular disease but its contribution to vascular remodelling and even its existence have recently been questioned.
- Tracking the fate of individual SMCs is difficult as no specific markers of migratory SMCs exist.
- This study used a novel, prolonged time‐lapse imaging approach to continuously track the behaviour of unambiguously identified, fully differentiated SMCs.
- In response to serum, highly‐elongated, contractile SMCs initially rounded up, before spreading and migrating and these migratory cells displayed clear phagocytic activity.
- This study provides a direct demonstration of the transition of fully contractile SMCs to a non‐contractile, migratory phenotype with phagocytic capacity that may act as a macrophage‐like cell.

**Abstract:**

Atherosclerotic plaques are populated with smooth muscle cells (SMCs) and macrophages. SMCs are thought to accumulate in plaques because fully differentiated, contractile SMCs reprogramme into a ‘synthetic’ migratory phenotype, so‐called phenotypic modulation, whilst plaque macrophages are thought to derive from blood‐borne myeloid cells. Recently, these views have been challenged, with reports that SMC phenotypic modulation may not occur during vascular remodelling and that plaque macrophages may not be of haematopoietic origin. Following the fate of SMCs is complicated by the lack of specific markers for the migratory phenotype and direct demonstrations of phenotypic modulation are lacking. Therefore, we employed long‐term, high‐resolution, time‐lapse microscopy to track the fate of unambiguously identified, fully‐differentiated, contractile SMCs in response to the growth factors present in serum. Phenotypic modulation was clearly observed. The highly elongated, contractile SMCs initially rounded up, for 1–3 days, before spreading outwards. Once spread, the SMCs became motile and displayed dynamic cell‐cell communication behaviours. Significantly, they also displayed clear evidence of phagocytic activity. This macrophage‐like behaviour was confirmed by their internalisation of 1 μm fluorescent latex beads. However, migratory SMCs did not uptake acetylated low‐density lipoprotein or express the classic macrophage marker CD68. These results directly demonstrate that SMCs may rapidly undergo phenotypic modulation and develop phagocytic capabilities. Resident SMCs may provide a potential source of macrophages in vascular remodelling.

## Key points

- Smooth muscle cell (SMC) phenotypic conversion from a contractile to a migratory phenotype is proposed to underlie cardiovascular disease but its contribution to vascular remodelling and even its existence have recently been questioned.
- Tracking the fate of individual SMCs is difficult as no specific markers of migratory SMCs exist.
- This study used a novel, prolonged time‐lapse imaging approach to continuously track the behaviour of unambiguously identified, fully differentiated SMCs.
- In response to serum, highly‐elongated, contractile SMCs initially rounded up, before spreading and migrating and these migratory cells displayed clear phagocytic activity.
- This study provides a direct demonstration of the transition of fully contractile SMCs to a non‐contractile, migratory phenotype with phagocytic capacity that may act as a macrophage‐like cell.

**Abbreviations**
AcLDL: acetylated low‐density lipoprotein
BSA: bovine serum albumin
CA: carotid artery
CCh: carbachol
EC: endothelial cell
FBS: fetal bovine serum
InsP_3_: inositol 1,4,5‐trisphosphate
PDGF‐BB: platelet‐derived growth factor‐BB
PE: phenylephrine
PV: portal vein
SM: smooth muscle
SMA: smooth muscle α‐actin
SMC: smooth muscle cell
SM‐MHC: smooth muscle myosin heavy chain
TMRE: tetramethylrhodamine

## Introduction

Atherosclerosis involves the focal build‐up of smooth muscle cells (SMCs) and macrophages under the endothelium in arteries (Ross, 1999). Macrophages may accumulate in the vascular wall because circulating monocytes adhere to the endothelium, migrate to the subendothelial space and differentiate into macrophages. These macrophage express scavenger receptors that facilitate the uptake of modified lipoproteins leading to cholesterol accumulation and the appearance of ‘foam cells’. Macrophage‐derived foam cells make up the fatty streak lesions that precede more advanced atherosclerotic plaques. However, in plaques, cells classified as macrophage (e.g. from CD68 expression) may also express proteins more usually associated with SMCs (Mietus‐Snyder *et al*. 2000; Allahverdian *et al*. 2014), e.g. SM α‐actin (SMA) and SM22α. In human coronary arteries, for example, 50% of foam cell‐rich lesions had co‐localisation of foam cell markers and SMA (Allahverdian *et al*. 2014). It has also been reported that human monocytes can undergo a transition to a SMA‐expressing myofibroblast‐like phenotype (Stewart *et al*. 2009). Thus, macrophage cells co‐expressing smooth muscle (SM) markers may be macrophage cells with SM markers or SM‐like cells with macrophage markers (Stewart *et al*. 2009; Ludin *et al*. 2012; Shen *et al*. 2012; Andreeva *et al*. 1997). Recent experimental observations have led to the proposal that SM may acquire a macrophage phenotype (Gomez *et al*. 2013; Allahverdian *et al*. 2014; Feil *et al*. 2014). The ability of contractile SMCs to dedifferentiate into a synthetic, migratory phenotype (known as phenotypic modulation) is unusual amongst differentiated cells and is thought to underlie vascular remodelling in atherosclerosis. However, the extent and even the existence of phenotypic modulation has recently been questioned (Holifield *et al*. 1996; Tang *et al*. 2012, 2013; Nguyen *et al*. 2013).

Many lines of evidence support the existence of phenotypic modulation though the data is indirect. For example, in arterial injury models, early ultrastructural studies showed cells that resemble SMCs in the fenestrae of the internal elastic lamina (Stemerman & Ross, 1972; Clowes *et al*. 1983), observations interpreted as evidence that SMCs were migrating from the media to the intima. More recently lineage tracing approaches have been used to study the origin of cells populating atherosclerotic plaques (Bennett *et al*. 2016). For instance, floxed LacZ reporter (encoding bacterial β‐galactosidase) apoE−/− mice with a tamoxifen‐inducible Cre recombinase knocked into one of the gene alleles of SM22α showed β‐galactosidase‐positive cells within plaques, evidence consistent with migration of SMCs to the plaque (Feil *et al*. 2004). Also using lineage tracing techniques, the SMC in atherosclerotic plaques were found to take on macrophage‐like features (Feil *et al*. 2014; Shankman *et al*. 2015), whilst by co‐staining 40% of the CD68‐positive cells in human coronary atherosclerosis expressed SMA (Allahverdian *et al*. 2014). These findings are each supportive of the existence of phenotypic modulation. On the other hand, in other lineage tracing studies using smooth muscle myosin heavy chain (SM‐MHC)‐Cre/LoxP‐enhanced green fluorescence protein (EGFP) mice, proliferative or synthetic SMCs were reported not to arise from the de‐differentiation of mature SMCs (Tang *et al*. 2012).

However, perhaps the major body of experimental work on phenotypic plasticity comes from studies in cultured cells, with extrapolation of these findings to processes that might occur during disease. Primary SMC cultures are either created from cells enzymatically dissociated from SM tissue or by the ‘explant method’, where an intact vessel is placed in culture and SMCs are assumed to grow out of the vessel wall. The cells derived from each of these methods are both migratory and proliferative – features consistent with phenotypic modulation.

Cultured SMCs, those cells identified in plaques and indeed cells tracked in linage tracing experiments are assumed to be derived from SM because of the markers expressed. Distinguishing the fully differentiated contractile phenotype of SM is relatively straightforward. Contractile SMCs have a highly elongated morphology, contract in response to neurotransmitters and hormones and express specific contractile proteins, notably SM‐MHC (Madsen *et al*. 1998; Sartore *et al*. 1999; Campbell & Campbell, 2012). On the other hand, the migratory phenotype is more poorly defined. In routine experimental practice, SMCs are identified by immunofluorescence using SM markers. SM‐MHC, the most specific SM marker, is not usually found in SMC cultures, its absence assumed to be a result of de‐differentiation. Instead, SMA and SM22α are probably the most widely used markers of the proliferative/migratory SM phenotype. However, SMA and SM22α are also expressed in many other non‐SMCs including endothelial cells (ECs), fibroblasts, monocytes and macrophage (Shapland *et al*. 1988; Arciniegas *et al*. 1992; Basson *et al*. 1992; Moroianu *et al*. 1993; Sartore *et al*. 2001; Martin *et al*. 2009; Ludin *et al*. 2012; Shen *et al*. 2012; Karagianni *et al*. 2013), raising significant uncertainties over cell sources.

The uncertainty is emphasised by previous findings that the bulk of growth in vascular disease (e.g. pathological lesions in atherosclerosis, restenosis and hypertension in humans, as well as neointimal thickening in injured vessels of experimental animals) is composed of cells with non‐muscle‐like characteristics (Glukhova *et al*. 1988; Campbell & Campbell, 1990; Leclerc *et al*. 1992; Pauletto *et al*. 1994). These cells had been thought to be SMCs which altered their protein expression during phenotypic modulation. However, cells derived from the vascular wall other than SMCs (e.g. progenitor cells) may be involved in plaque growth (Bochaton‐Piallat *et al*. 1996; Holifield *et al*. 1996; Z. Li *et al*. 1997, S. Li *et al*. 2001; Hao *et al*. 2002; reviewed by Wang *et al*. 2015) and SMCs have been reported to be incapable of altering phenotype either *in vitro* or *in vivo* (Holifield *et al*. 1996; Tang *et al*. 2012), with the proposal that all cells studied in culture are derived from sources other than SM (Tang *et al*. 2012, 2013).

The ability of SM to undergo phenotypic modulation, including adopting macrophage‐like characteristics, has significant implications for our understanding of atherosclerosis and plaque development. However, ongoing doubts and potential confusion in the identity of the cells weakens confidence in the proposal. Therefore, in this study we sought to directly demonstrate whether or not fully differentiated, contractile SMCs are capable of undergoing phenotypic modulation and taking on a macrophage‐like phenotype. To provide an unambiguous, direct demonstration of resulting phenotypic changes, we established high‐resolution, simultaneous phase contrast/fluorescence time‐lapse microscopy to track in detail the fate of individual, freshly isolated, fully differentiated SMCs. Unambiguously identified SMCs from four very different sources (carotid artery (CA); descending aorta; portal vein (PV); distal colon), including two (CA and aorta) that are common sites of atherosclerosis, were used to determine whether SMCs from different tissues underwent the same phenotypic modulation process. The SMCs were imaged continuously during their first days in standard, widely used culture conditions. Freshly dissociated SMCs are readily identified by their unique elongated spindle‐shape and their pronounced contractile responses to phenylephrine (PE; vascular) or carbachol (CCh; gastrointestinal). Their distinctive morphology (there are no other cells with this morphology in the isolate) and functional properties provide an unequivocal identification of SM. In previous work, we have established that these elongated cells, which stain for SM‐MHC, exhibit the electrical and contractile behaviour expected from SMCs (McCarron & Muir, 1999; Rainbow *et al*. 2009; Olson *et al*. 2012). Only cells unambiguously identified as SMCs were tracked in the present study. The results provide definitive evidence that fully contractile SMCs can rapidly undergo phenotypic modulation. The resulting migratory SMCs are highly dynamic and may directly communicate with nearby cells. Significantly, we also show that migratory SMCs display clear phagocytic behaviour, including the ability to phagocytosis cell fragments and fluorescent microbeads. These results suggest that SMC phenotypic plasticity exists and SM could potentially behave as a resident vascular macrophage.

## Methods

### Ethical approval

All experiments were carried out on freshly dissected tissue from animals not subject to any other treatments. Killing was in accordance with UK regulations (Animals (Scientific Procedures) Act 1986, revised under European Directive 2010/63/EU). Male Sprague‐Dawley rats or Dunkin Harley guinea‐pigs were killed by trained technicians with an intraperitoneal overdose of sodium pentobarbital (Euthatal, 200 mg kg^−1^).

### Materials and solutions

Unless otherwise noted, all reagents were purchased from Sigma‐Aldrich (Dorset, UK). Cell culture media was obtained from Life Technologies (Paisley, UK), as were Fluo4‐AM, 1.0 μm yellow‐green fluorescent polystyrene microspheres, AlexaFluor488‐labelled acetylated low‐density lipoprotein (AcLDL), tetramethylrhodamine (TMRE) and CellLight Histone 2B‐GFP. The enzymes used for cell isolation were collagenase Type F (Sigma Aldrich), collagenase Type 3 (Worthington, NJ, USA), papain (Worthington) and hyaluronidase (Sigma Aldrich). Cell culture dishes with gridded glass (Grid‐500 μDish), hydrophilic plastic (ibiTreat) and collagen IV coated substrates were purchased from Ibidi (Germany). The antibodies used for immunocytochemistry were: mouse anti‐SMA‐Cy3 (C6198, Sigma‐Aldrich), goat anti‐SM‐MHC (MYH11) (sc‐79079, Santa Cruz, TX), mouse anti‐CD68 (ab955, AbCam, UK), sheep anti‐von Willebrand Factor‐FITC (ab8822, AbCam), donkey anti‐goat‐AlexaFluor488 (A11055, Life Technologies) and donkey anti‐mouse‐AlexaFluor555 (A31570, Life Technologies).

The buffers used were: Mops (145 mm sodium chloride, 2 mm MOPS, 4.7 mm potassium chloride, 1.2 mm monosodium phosphate, 5 mm glucose, 0.02 mm EDTA, 2 mm sodium pyruvate, 1.2 mm magnesium chloride, 2 mm calcium chloride, pH 7.4); isolation buffer, with or without 2 mg ml^−1^ fatty acid free bovine serum albumin (BSA) (80 mm sodium glutamate, 55 mm sodium chloride, 6 mm potassium chloride, 10 mm glucose, 10 mm Hepes, 1 mm magnesium chloride, 0.1 mm calcium chloride, 0.2 mm EDTA, pH 7.4); and bath solution (80 mm sodium glutamate, 40 mm sodium chloride, 20 mm tetraethylammonium chloride, 1.1 mm magnesium chloride, 3 mm calcium chloride, 10 mm Hepes, 30 mm glucose, pH 7.4).

### Cell isolation

Tissues were removed from male guinea‐pigs (∼500 g) and rats (250–300 g) and were immediately placed into Mops buffer. SMCs were freshly isolated from the media/muscularis of PV, CA, distal colon and descending aorta using methods similar to those previously reported (Kamishima & McCarron, 1998; Bradley *et al*. 2003; Chalmers *et al*. 2012).

To prepare PV tissue, the adventitia and surrounding connective tissue were carefully cut away under a dissecting microscope and the vessel denuded of endothelium. However, it was not possible to fully remove the adventitia from CA by dissection alone. Instead, similar to Gonzalez *et al*. (2001), an intact CA (tied off at the ends) was incubated for 30 min at 37°C in 2 mg ml^−1^ Type 3 collagenase. The adventitia could then be readily removed from the vessel using two pairs of fine tweezers to pull the adventitia away from the vessel, which was then cut open and denuded of endothelium. The aorta was prepared similarly but with a 50 min collagenase incubation. Colonic tissue was prepared by opening and pinning out the colon, first cutting away the mucosa before turning the tissue and carefully removing the serosa.

SMCs were isolated from the prepared tissue by enzymatic digestion and trituration. All digestions were at 34.5°C, with enzymes diluted in BSA‐containing isolation buffer and the tissues washed with the same buffer after each enzyme incubation. PV tissue was incubated in 2.2 mg ml^−1^ Type F collagenase with 1.0 mg ml^−1^ hylauronidase for 15 min followed by 1.7 mg ml^−1^ papain with 0.7 mg ml^−1^ dithioerythritol for 15 min. CA and aortic tissues were incubated similarly but for 30 min in each solution. Colon tissue was incubated first in 1.0 mg ml^−1^ papain with 0.7 mg ml^−1^ dithioerythritol for 25 min and secondly in 2.5 mg ml^−1^ Type 3 collagenase for 25 min. To release SMCs, tissue was washed three times with sterile BSA‐free isolation buffer and triturated in a sterile environment with fire‐polished glass pipettes.

Macrophages were isolated from the peritoneal cavity by cutting away the abdominal skin to expose the peritoneal wall. Ice‐cold, sterile PBS was then injected into the cavity until the abdomen inflated, and the abdomen massaged for ∼2 min. A small incision was then made in the peritoneal wall and the peritoneal fluid aspirated with a Pasteur pipette. An aliquot of the collected cells was left to settle in glass‐bottomed dish at 4°C before fixing and staining.

### Cell culture

Freshly isolated SMCs were seeded (∼5 × 10^4^ cells) into a gridded glass chamber and were cultured in 1:1 Waymouth's:Ham's F‐12 media containing 10% fetal bovine serum (FBS) with 1% penicillin–streptomycin and 1% l‐glutamine at 37°C in 5% CO_2_ and 80% humidity. For tracking bead uptake, 1 μm yellow‐green fluorescent polystyrene microspheres were washed three times in media, opsonised in 50% FBS for 30 min at 37°C and added to the culture media to give a concentration of 1–2 × 10^6^ beads ml^−1^. Before assessing bead uptake, cells were washed three times to remove any loosely bound beads. AlexaFluor488‐labelled AcLDL was added to cultures at 10 μg ml^−1^, whilst TMRE was used at a 20 nm and CellLight Histone 2B‐GFP at ∼25 particles per cell.

When the contractility of individual SMCs was first confirmed prior to culturing, SMCs were loaded into a culture dish in either bath solution or serum‐free media and left to settle. An InsP_3_‐generating agonist was then puffed (see below) onto the SMCs of interest. After allowing the SMCs to relax, serum‐containing media was washed into the dish (when using buffer) or an aliquot of serum pipetted into the dish (when using serum‐free media) and recording and incubation then proceeded as normal. As the dish was exposed to the room environment during puffing, to ensure sterility extra media changes were carried out (typically around 1–3 h and 24 h after starting culturing) and the media then changed every 2–3 days as normal.

### Microscopy and image analysis

To track SMC fate, a customised wide‐field fluorescence with simultaneous phase contrast imaging system was used. This was based around an inverted Ti‐E microscope with Perfect Focus System (Nikon, UK) to correct for focus drift during long‐term imaging and was equipped with a pE100 white LED light source (CoolLED, UK) for bright‐field/phase contrast imaging, a DeltaRAM X monochromator with 75 W xenon lamp for fluorescence imaging (Photon Technology International, UK) and an iXon888 EMCCD camera (Andor, Northern Ireland) for image capture. A microscope stage‐top incubator (Okolab, Italy) was used to maintain the cells at 37°C and 5% CO_2_.

The system allowed for the acquisition of simultaneous bright‐field/multiwavelength fluorescence time‐lapse imaging and was controlled by WinFluor software (Strathclyde Imaging Software, UK), adapted to allow either continuous time‐lapse or time‐lapse interspersed with rapid bursts recording modes. Images were captured every 30 s during time‐lapse recordings and with a frame rate of 5–10 Hz for bursts. For 3D reconstruction microscopy, *z*‐stacks (100 nm step size) were acquired using a P‐725 PIFOC Long‐Travel Objective Scanner and E‐665 Piezo Amplifier/Servo Controller (Physik Instrumente, Germany) mounted on a TE2000 microscope (Nikon) controlled by WinFluor.

All image processing was carried out using Image‐Pro Analyser 7.0 with SharpStack Plus deconvolution plug‐in (Media Cybernetics, MD, USA). For relative quantitation of antibody staining, all samples were imaged at the same time using the same the recording conditions and images processed using the same operations. Only healthy, non‐overlapping cells were measured and the focal plane was adjusted to the maximum intensity for the SMC being imaged (freshly isolated SMCs being relatively deep, with a diameter of 5–10 μm). Images were background and flat‐field corrected and filtered with a 3 × 3 median filter (to minimise the contribution of any spurious high intensity pixels). The maximum intensity was then measured for each SMC.

### Bead uptake quantification

Bead uptake in aortic SMCs was assessed by culturing SMCs from adventitia‐stripped vessels in a gridded chamber for 1 week. Opsonised fluorescent microbeads were then added to give 4 × 10^6^ beads ml^−1^ and the cultures incubated for a further 24 h, before washing three times to remove any loosely bound beads prior to fixing. Images of individual grid squares were acquired (>7 squares per culture, all squares with a confluency of <50%) and the total number of beads taken up by each individual SMC imaged was counted (>150 cells for each culture; 3 cultures each from different animals).

### Ca^2+^ imaging and agonist application

For fluorescent Ca^2+^ imaging, cells were loaded with 10 μm Fluo4‐AM (Life Technologies, UK) and washed before imaging in either bath solution or media supplemented with CaCl_2_ (3 mm total calcium). PE or CCh was puffed onto a cell (250–500 μm, 5–10 s) via a pulled glass pipette using a hydrostatic pressure ejection PicoPump system (WPI, FL, USA). Platelet‐derived growth factor‐BB (PDGF‐BB) was applied similarly (200 nm). Relative changes in fluorescence intensity (*F*/*F*_0_) were measured as the mean intensity of a region within the cell body normalised to the intensity measured prior to puffing/adding FBS.

### Immunocytochemistry

Cells were fixed in 10% formalin, quenched with 100 mm glycine, permeabilised with 0.1% Triton X‐100 and blocked using 2% BSA in PBS or 2% donkey serum when staining for SM‐MHC. Antibody incubations were at room temperature for 1 h.

### Data analysis

Data analysis was performed in OriginPro9.0 and Minitab17. All values are quoted as means (standard deviation; sample size) except for data on marker expression, where values are quoted as medians with the range of 1st–3rd quartile values. A non‐parametric Mann‐Whitney test with *P* < 0.05 was used for the latter to determine whether there was a statistical difference between native and cultured populations.

## Results

### Fully contractile SMCs were isolated from the media/muscularis of freshly dissected tissue

**Figure 1:**
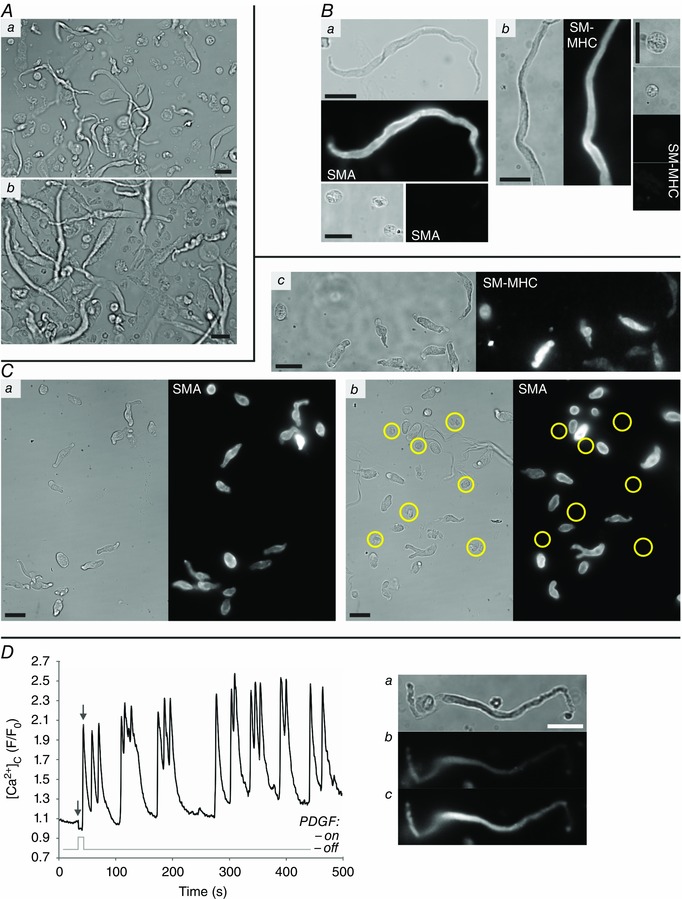
**Native SMCs** *A*, freshly isolated cell suspensions obtained from the tunica media of PV (*Aa*) and the muscularis externa of colon (*Ab*) guinea‐pig tissue showing the range of cell types present. *B*, SMA and SM‐MHC staining of freshly isolated SMCs, which have a unique highly elongated morphology, and round cells that are not of SM origin (*Ba* cells from PV; *Bb* cells from colon). *C*, freshly isolated cells from rat CA, both from vessels fully stripped of adventitia (*Ca, Cc*) and a vessel with adventitia remaining (*Cb*). In the later a number of cells that do not stain for SM markers were present (yellow circles); in the former all cells stained for both SMA and SM‐MHC, whether fully elongated or not. *D*, the highly oscillatory [Ca^2+^]_c_ response of a PV SMC (*Da*) to a single 10 s puff of PDGF‐BB as measured by changes in mean Fluo‐4 fluorescence for a region of interest within the cell body (*Db*, immediately before puffing, corresponding to the first grey arrow marked on the graph; *Dc*, corresponding to the initial peak following PDGF puffing, second grey arrow on graph). All scale bars are 25 μm.

**Figure 2:**
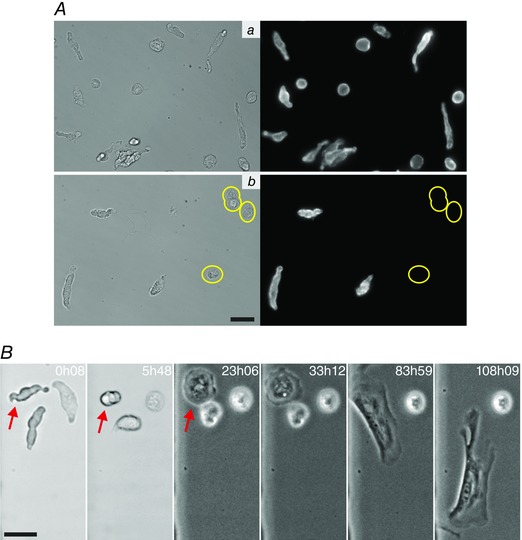
**Freshly isolated aortic SMCs and their phenotypic modulation** *A*, freshly isolated cells from the descending rat aorta stained for SMA. When the aortic tissue is fully stripped of adventitia and endothelium (*Aa*), all cells in the isolation stain for SM markers, even those with a more rounded morphology. In contrast, when the adventitia is not completely removed (*Ab*), non‐SM cells that do not stain for any SM markers are present (circled in yellow). *B*, a time sequence showing the change that a tracked aortic SMC (indicated by red arrow in initial frames) undergoes as it transforms in culture from its native, contractile state to a migratory phenotype. In this example the SMC became migratory from ∼65 h onwards. The times marked in the images (in hours and minutes) are the length of time in culture. All scale bars are 25 μm.

**Figure 3:**
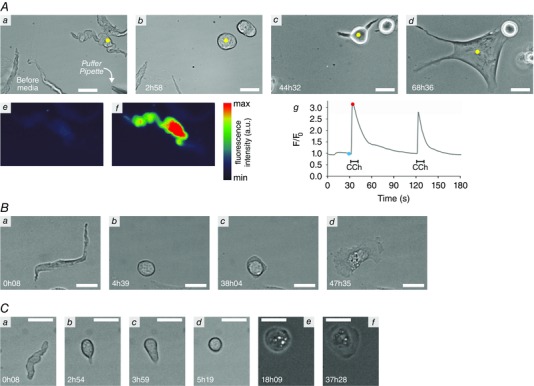
**Phenotypic modulation of SMCs in culture** Time sequences showing the changes that SMCs isolated from colon (*A*), PV (*B*) and CA (*C*) undergo as they transform from their native, highly elongated phenotype (*Aa, Ba, Ca*) to a fully spread morphology typical of cultured cells (*Ad, Bd, Cf*). The SMCs are initially fully contractile, displaying strong InsP_3_‐evoked [Ca^2+^]_c_ signals as measured by Fluo‐4 fluorescence (*Ae–g* shows the [Ca^2+^]_c_ response from the native SMC tracked in *Aa–d*; *Ae*, before puffing CCh, corresponding to blue dot in *Ag*; *Af*, upon puffing CCh, red dot in *Ag*; *Ag*, relative change in measured fluorescence following two CCh puffs). In response to culture conditions, the SMCs rounded up fully (*Ab, Bb, Cd*) before starting to spread (*Ac, Bc, Ce*) outwards, either by putting out elongated processes or through lamellipodia spreading in all directions. CA cells often partially adhered to the substrate prior to rounding up (*Cb, Cc*). The sequences in this figure correspond to Movies 1–3 in Supporting information and the times marked in the images (in hours and minutes) are the length of time in culture. Scale bars 25 μm.

Cell isolations from the SM layer of CA, aorta, PV or colon from either rat or guinea‐pig contained highly elongated SMCs that stained strongly for both SM‐MHC and SMA (Figs 1 *A–C* and 2 *A*). The cells were fully contractile in response to a pulse of InsP_3_ (inositol 1,4,5‐trisphosphate)‐generating agonists (PE or CCh), as shown in Figs 3 *A* and 8 *A*, and Movies 1 and 7 in Supporting information. The cells also responded strongly to PDGF with varying forms of oscillatory fluctuations in cytoplasmic calcium concentration ([Ca^2+^]_c_) (Fig. 1 *D*). The [Ca^2+^]_c_ changes resulting from a single short duration (5–10 s) puff of PDGF lasted long (>5 min) after the stimulus had ended (Fig. 1 *D*). However, with PV and colon, cell types other than SMC were also present in the isolations (Fig. 1 *A*), even after careful removal of the adventitia/serosa and endothelium/mucosa. These cells were not SMCs – they did not stain for any SM markers (Fig. 1 *B*) – and in culture they rapidly spread and became migratory. The presence of these non‐SMCs means that, to be certain the individual cells under observation were SMCs, continuous tracking from their native, elongated state was essential. In contrast to the results with PV and colon, when the adventitia was completely stripped from CA and aorta, using an additional enzymatic digestion step (based upon the method of Gonzalez *et al*. 2001), the resulting cell isolations contained SMCs alone; all cells stained for SMA and SM‐MHC (Figs 1 *Ca* and *c* and 2 *Aa*). When the adventitia was not fully removed from CA and aorta tissue (e.g. with mechanical dissection alone), cells other than SMCs were also found in the isolations (Figs 1 *Cb* and 2 *Ab*).

### SMCs readily undergo phenotypic modulation following exposure to serum‐containing culture medium

Freshly isolated cells were seeded in a gridded glass chamber, so that the specific tracked cells could be easily identified following removal from the microscope (e.g. after media changes), and were cultured in media containing 10% FBS. Tracking of individual SMCs by time‐lapse microscopy began immediately after the addition of media. Under the standard culture conditions employed, all SMCs tracked by time‐lapse microscopy, irrespective of their tissue source, rapidly altered their phenotype when exposed to serum‐containing media. A consistent sequence of changes occurred, as illustrated in Figs 2 *B* and 3 and Movies 1–3 in Supporting information. During the first few hours in culture, the initially elongated SMCs rounded up (Fig. 3 *Ab*, *Bb* and *Cd*), though significant tissue‐tissue and cell–cell variation was observed in timing. Not all the SMCs observed reached or survived beyond this stage, with apoptosis often occurring early in the transformation process (apoptosis was inferred from distinct changes observed when imaging in brightfield, such as cells becoming more transparent with less distinct cell boundaries, accompanied by noticeable blebbing and ultimately resulting in completely immobile cells). Apoptosis was particularly common in arterial cells, with only 10 of 67 tracked CA SMCs remaining viable after 72 h (an example of a cell undergoing apoptosis can be seen in Movie 3 in Supporting information alongside the tracked, viable cell). After remaining rounded for periods of time that varied from 2 h up to 3 days, the SMCs spread outwards either by extending narrow, elongated processes from small regions of the rounded cell body (Fig. 3 *Ac*) or by projecting lamellipodia outwards in all directions (Fig. 3 *Bc* and *Ce*).

### Spontaneous contractions occur during phenotypic modulation in SMCs from PV and colon but not CA

**Figure 4:**
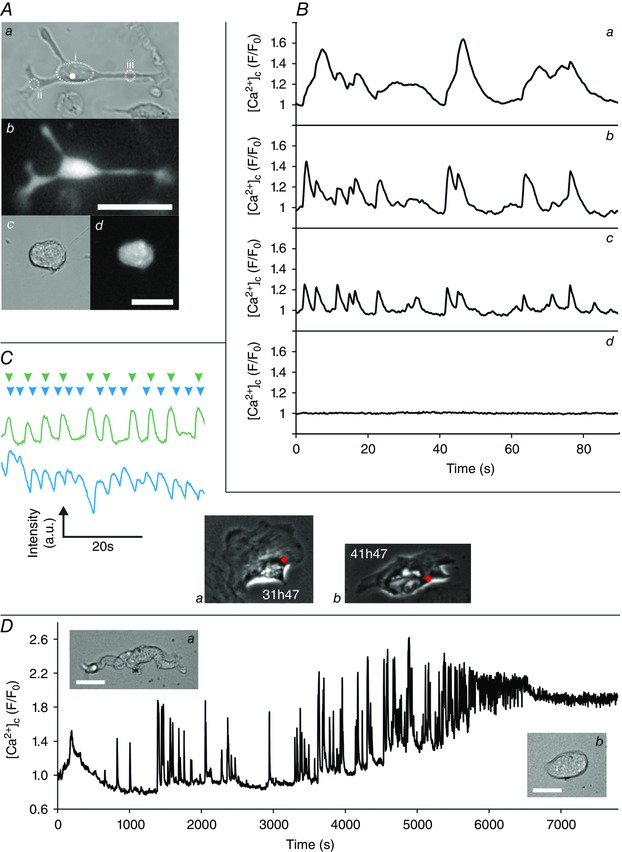
**Repetitive contractions and [Ca^2+^]_c_ oscillations observed during phenotypic modulation** *A*, a PV SMC that displayed spontaneous contractions 48 h after being placed into culture (*Aa*, brightfield; *Ab* Fluo‐4 fluorescence). Spontaneous contractions were accompanied by oscillations in [Ca^2+^]_c_ (*B*), with varying spatiotemporal signals throughout the cell (*Ba–c* correspond to the mean Fluo‐4 intensity measured in the regions highlighted in *Aa*). Spontaneous [Ca^2+^]_c_ oscillations were not observed for fully rounded cells (*Ac*, brightfield; *Ad*, Fluo‐4, *Cd*, mean whole‐cell fluorescence). *C*, spontaneous contractions can be monitored by measuring the changing intensity of a region on a phase contrast recording as adjacent dark and light subcellular areas moves into and out of the region during contraction. Examples of traces from the same cell at two different times (green intensity trace corresponds to region marked by the red dot in *Ca*; blue trace to the red dot in *Cb*; arrowheads above the traces mark the approximate time of individual contractions) which, after reaching a maximum rate of ∼11 ‘beats’ per minute for strong contractions (green), showed a decrease in contraction strength but an increase in contraction rate (blue). *D*, strong [Ca^2+^]_c_ fluctuations were observed during the initial transition from an elongated contractile cell to a rounded cell (fluctuating mean whole‐cell [Ca^2+^]_c_ levels during the first 2 h in culture; inserts *Da* and *Db* show the PV SMC morphology at the beginning and end of the trace, respectively). The spontaneous contractions described in *A–C* can be seen in Movie 4 in Supporting information. Scale bars are 25 μm.

As they began to spread, all tracked SMCs from PV and colon underwent a period of spontaneous, repetitive contractions (Movie 4 in Supporting information). Typically, these contractions occurred over a period of 12–24 h as the cell spread outwards over the substrate and reached a maximum of 10 (0.6; *n* = 4) ‘beats’ per minute. These contractions were accompanied by spontaneous oscillations in [Ca^2+^]_c_ (Fig. 4 *A* and *B*). In most cases the contraction rate decreased after reaching its maximum value but on occasion, instead of the rate diminishing, the strength of the contractions progressively weakened whilst the rate increased until the contractions faded away (Fig. 4 *C*, Movie 4 in Supporting information). Interestingly, SMCs isolated from CA and aorta were not observed to undergo this sustained period of repetitive contractions. Nor did oscillations in [Ca^2+^]_c_ occur when the SMCs were fully round (Fig. 4 *Bd*): whilst strong fluctuations were observed after initial exposure to serum and continued as the cell began to round up (Fig. 4 *D*), [Ca^2+^]_c_ stabilised as the cell became fully round.

### Modulated SMCs are highly dynamic and actively involved in direct communication with nearby cells

**Figure 5:**
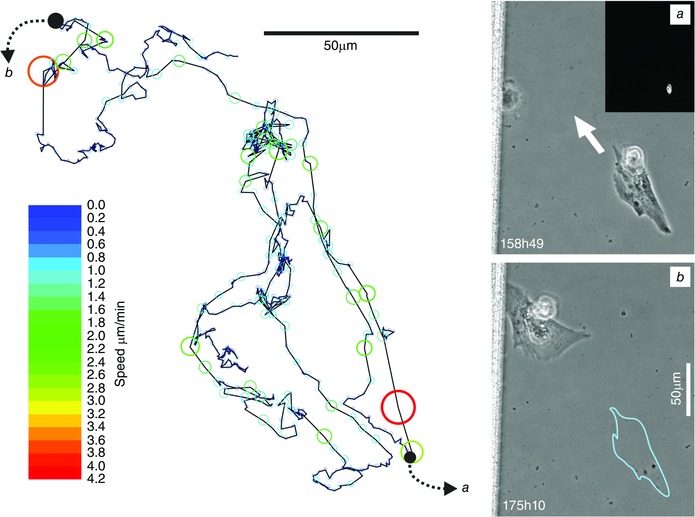
**Tracking the movement of a motile SMC** The motion of a colonic SMC over a 3 day period tracked via the signal obtained from its fluorescently labelled nucleus. Images *i* (inset shows Histone 2B‐GFP) and *ii* correspond to the cell at the positions marked on the motion track by black dots (blue outline in *ii* shows cell position in *i*). Automated tracking was performed using Image Pro Analyser and the size of the overlaid bubbles on the motion track corresponds to the instantaneous speed of the cell, with the colour mapped to the speed scale bar. This image corresponds to Movie 5 in Supporting information.

After spreading fully, the SMCs displayed a completely transformed morphology (Figs 2 *B* and 3 *Ad*, *Bd*, *Cf*) and became highly dynamic. The overall phenotypic modulation process was similar whether the SMCs were cultured on glass coverslips, tissue culture plastic or collagen IV‐coated substrates, as well as when using different culture media (1:1 Ham's F‐12:Waymouth's, DMEM or 1:1 DMEM:Ham's F‐12, data not shown). Nearly all the tracked SMCs became motile, exploring nearby regions of the substrate (Fig. 5, Movie 5 in Supporting information) with a typical mean velocity of 0.5 (0.1; *n* = 4) μm min^−1^ for colon cells. PV cells was slightly slower at 0.4 μm min^−1^. These speeds are similar to that reported for fibroblasts. Motion tracking was performed using the fluorescent signal obtained from nuclear labelling by transduction with the Histone 2B‐GFP CellLight reagent. SMCs only expressed such fluorescent fusion proteins after they had spread (even when the reagent was added to the culture media at the outset).

**Figure 6:**
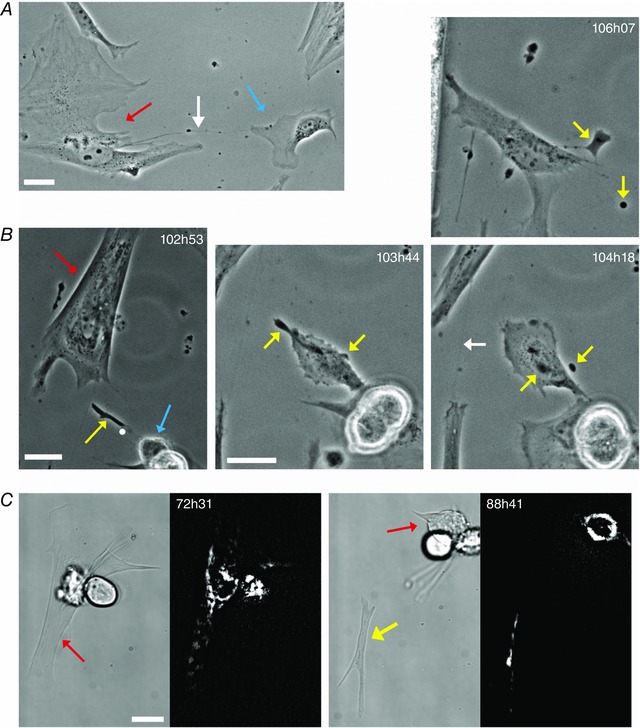
**Cell‐cell communication in modulated SMCs** *A*, an example of a TNT‐like structure (white arrow) connecting a tracked PV SMC (red arrow) to a nearby non‐SMC (blue arrow). *B* (102h53–106h07), a subcellular structure (yellow arrow, 102h53) extruded by a tracked colonic SMC (red arrow). This extruded structure subsequently interacts with the daughter cell of a nearby dividing cell (blue arrow). The white dot in the first image (102h53) corresponds to the centre of the 103h44 image, which shows one of the cells interacting with the extruded structure. The extruded structure subsequently fragments into two parts (yellow arrows 104h18). After this fragmentation, the daughter cell casts off both fragments (106h07, where the larger fragment remains connected to the cell by a TNT‐like structure) before it migrates onwards. The larger of the two fragments is eventually engulfed by another cell. A second example of a SMC TNT can also be seen at 104h18 (white arrow). *C*, an example of extrusion from a tracked colonic SMC (red arrow) of a larger, microplast‐like cell fragment (yellow arrow). The fluorescent images show mitochondrial staining with TMRE and demonstrate that the extruded fragment contains a number of polarised mitochondria. The SMC did not round up prior to pinching off this cellular fragment; rather it underwent a series of strong contractions. Following extrusion, no overall movement of the fragment was observed during the following 56 h, after which the fragment was picked up and carried off by another cell. All scale bars are 25 μm.

The migratory SMCs displayed highly dynamic cell–cell communication behaviours involving the exchange of cellular material. Two types of communication occurred. First, they were observed forming long, fine cellular processes (so‐called tunnelling nanotubes) that formed direct connections with other nearby cells (Fig. 6 *A*). Secondly, they frequently extruded cellular fragments (Fig. 6 *B*), typically shedding 1–10 μm sized extracellular bodies, but occasionally pinching off larger microplast‐like structures (Fig. 6 *C*). These extracellular bodies, which may contain various cellular components including mitochondria (as in Fig. 6 *C*), could subsequently interact with or be ingested by a nearby cell. Even those few cells that did not move significantly from their initially spreading point still displayed these highly dynamic forms of communication.

### As SMCs become motile there is a concomitant loss of response to some InsP_3_‐generating agonists

**Figure 7:**
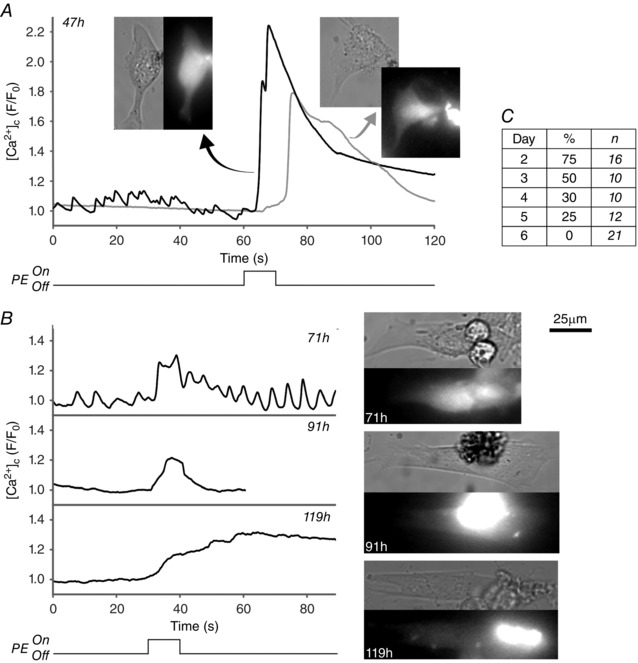
**Loss of response to the InsP_3_‐generating agonist PE as PV SMCs undergo phenotypic modulation** Changes in [Ca^2+^]_c_ in response to PE puffing were measured by relative changes in Fluo‐4 fluorescence for PV SMCs that were maintained in culture conditions for 2–6 days. *A*, example traces showing a strong [Ca^2+^]_c_ response to PE obtained from two PV SMCs after 47 h in culture (inset images are brightfield and Fluo‐4 fluorescence). Responses declined from day 3 onwards (*B*) along with a decrease in the overall percentage of cells responding to PE (*C*). Cells were counted as a ‘responder’ if an increase in *F*/*F*_0_ of > 1.1 occurred. Fluorescence intensity values were measured from a circular region of interest within the cell body (with an expanded region of interest to account for cell contraction where necessary). The traces shown for 47 h and 119 h correspond to the cells in Movie 6 in Supporting information.

To determine whether the gain of dynamic cell behaviours is associated with a remodelling of Ca^2+^ signalling processes, the ability of SMCs to respond to InsP_3_‐generating agonists with a rise in [Ca^2+^]_c_ was measured over their first few days in culture as the cells underwent phenotypic modulation. PE was puffed daily onto individual PV SMCs from days 2–6 in culture and the resulting changes in [Ca^2+^]_c_ measured fluorescently (Fig. 7). After 47 h in culture, 75% of the SMCs tested responded with a clear change in [Ca^2+^]_c_ that was significantly larger than any of the aforementioned spontaneous oscillations (as seen in Fig. 7 *A*). At this time point (47 h), 67% of the cells responding also contracted strongly in response to the PE puff (with significantly stronger contractions than the spontaneously occurring ones).

This ability of the SMCs to contract in response to PE was largely lost from day 3 onwards, with only one cell observed to contract after day 2 (see Movie 6 in Supporting information) and then with a slower contraction and [Ca^2+^]_c_ rise and a lower peak [Ca^2+^]_c_. Similarly, from day 3 onwards (Fig. 7 *B*) there was a steady decline in the number of SMCs responding with an increase in [Ca^2+^]_c_ (Fig. 7 *C*) and peak [Ca^2+^]_c_ amplitudes were noticeably lower than those obtained on day 2 (whilst peak *F*/*F*_0_ values were ≥1.6 for 58% of SMCs on day 2, all cells from day 3 onward had peak values ≤1.4). By day 6, the response to PE was completely lost, with no SMCs responding. Similar results were obtained with cultured colon SMCs when puffing the InsP_3_‐generating agonist CCh (data not shown).

### Fully contractile SMCs can rapidly remodel into a cell capable of phagocytosis

**Figure 8:**
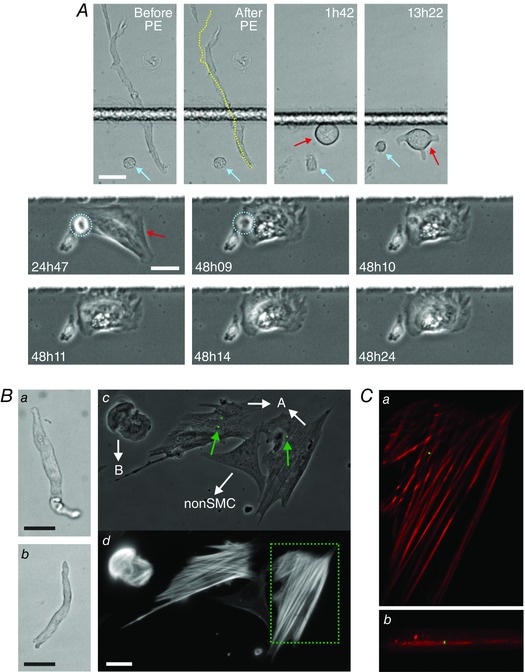
**Phagocytic behaviour of tracked PV SMCs** *A*, a PV SMC that contracted in response to PE puffing (compare cell length in Before and After PE images, yellow line in latter being cell mid‐line from Before PE) was tracked continuously as it transformed in culture (length of time in culture is noted in each image, SMC tracked is marked by red arrow). A cell that was not a SMC but was also isolated from the media layer was present in the field of view (blue arrow). The non‐SMC initially spread and migrated before re‐rounding (upper row images). Around 38 h the non‐SMC (circled in blue in the middle row) underwent apoptosis (cell became immobile, with signs of blebbing and a rapid change in cell contrast), shortly after which it was engulfed by the spreading SMC (48h09–48h24). This phagocytosis event can be seen in more detail in Movie 7 in Supporting information. *B*, the uptake of fluorescent microbeads by modulated PV SMCs. Two freshly isolated PV cells (*Ba* and *Bb*) were tracked after being placed into culture. Both SMCs spread, became motile and began to engulf extracellular debris, with the cell in *Ba* dividing at 72 h (daughter cells are indicated by the white arrows pointing towards A in *Bc*; cell *Bb* corresponds to B in *Bc*). Fluorescent microbeads were introduced into the culture at 98 h and the SMCs appeared to internalise microbeads from ∼101 h onwards, after which they were washed, fixed and stained (at 118h30). *Bc* shows the microbead fluorescence (green, beads indicated by green arrows) overlaid on a phase contrast image of the fixed cells. *Bd* shows the SMA staining corresponding to *Bc* (there is a cell in the field of view that is not of SM origin and does not stain for SMA). *C*, image planes from a deconvolved *z*‐stack (microbeads in green, SMA in red), corresponding to the area marked by the dotted box in *Bd*, show that the bead was in the same focal plane as the inner actin filaments, confirming its internalisation (*Ca*, the *x–y* plane corresponding to the centre of the microbead; *Cb*, an *x–z* maximum intensity projection). All scale bars are 25 μm.

After undergoing phenotypic modulation, tracked SMCs from all three tissue sources were frequently observed phagocytosing extracellular debris, a radical change in functional behaviour of the cell. An example of this can be seen in Movie 5 in Supporting information, in which a tracked colonic SMC can be seen to engulf a large fragment of cellular debris. Another example of the phagocytosis of extracellular debris can be seen in Movie 2 in Supporting information. Phagocytosis can even occur early in the phenotypic modulation process. Figure 8 *A* and Movie 7 in Supporting information show a PV cell whose contractility was first confirmed by PE puffing before the same cell was tracked during its first days in culture. After just 48 h, the recently contractile SMC phagocytosed a nearby cell that had undergone apoptosis. This was not an isolated behaviour; the majority of SMCs tracked appeared to phagocytose extracellular material.

**Figure 9:**
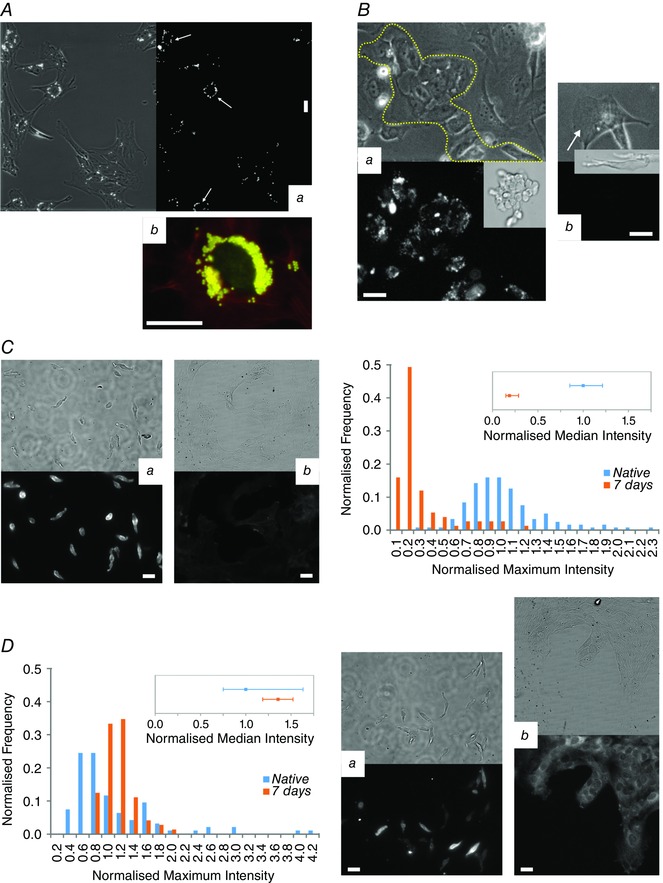
**AcLDL uptake and SM marker expression in native and cultured SMCs** *A*, examples of aortic SMCs phagocytosing microbeads. In *Aa* almost all SMCs have phagocytosed ≥1 bead (bead fluorescence on right), with some cells having phagocytosed ≥15 beads, converging them in the region around the nucleus (white arrows). Occasionally, SMCs internalised very large numbers of beads (*Ab*, > 180 beads; beads yellow, SMA red, nucleus green). *B*, ECs readily took up AcLDL (the images in *B* correspond to Movie 9 in Supporting information). *Ba* shows a tracked patch of ECs marked by the dotted line, although some endothelial cells have broken away from the main patch (EC patch in its native state shown in the inset). The fluorescent image (right side) shows clear AlexaFluor488‐AcLDL uptake. However, SMCs in the same culture did not take up AcLDL (*Bb* and *Bc* show tracked SMCs, indicated by white arrows with the fluorescent intensity range the same as in *Aa* and insets showing the SMCs when freshly isolated). *C*, SMA expression in freshly isolated SMCs (*Ca*) was significantly higher (*P* < 0.05) than in SMCs cultured for 1 week (*Cb*, the fluorescence image has the same intensity range as *Ca*). The histogram (*C*, right panel) shows summarised data from the measured maximum intensities of 119 native cells and 75 cultured cells (imaged at same time with the same settings and all images median filtered), with the intensity being normalised to the median value for the native cells and the frequency to the total number of cells. *D*, in contrast, SM‐MHC fluorescence was not reduced (*Da* native, *Db* cultured), as shown in the histogram (*D*, left‐panel; data was processed as in *C* for 94 native cells and 72 cultured cells). Note the decrease in the quartile range from native to 1 week and the presence of strongly stained cells in *Da* that are not present in *Db*. All scale bars are 25 μm.

To better quantify the phagocytic behaviour and to confirm that SMCs were truly internalising foreign material, opsonised 1.1 μm diameter fluorescent microbeads were introduced into cultures; the uptake of microbeads being a standard assay for macrophages. Firstly, microbeads were introduced into cultures with motile SMCs that had been tracked continuously from their native state. By fixing the SMCs following microbead phagocytosis (Fig. 8 *B* and Movie 8 in Supporting information, which shows examples of bead uptake) and performing 3D reconstruction microscopy on the fixed SMA‐stained cells, microbead internalisation was confirmed. (SMA staining was used to identify intracellular focal planes; beads in the same focal planes are therefore intracellular. It was not used for SMC identification, as the SMCs had been tracked continuously from their native state.) The colon SMC bead phagocytosis in Movie 8 in Supporting information (which also shows bead phagocytosis by a PV SMC) is a continuation of the tracking in Fig. 3 *A* and Movie 2 in Supporting information where SMC contractility was initially confirmed by CCh puffing. Together these results demonstrate that a fully differentiated SMC can indeed adopt a phagocytic phenotype. Secondly, to quantify uptake, microbeads were added to SM cultures from adventitia‐stripped aorta. As discussed above, these isolations contain only SMCs (Fig. 2 *A*), allowing for the analysis of larger numbers of SMCs without tracking. After a 24 h incubation and following thorough washing, imaging of the cultures (Fig. 9 *A*) showed that 70% (18%; *n* = 3 animals, >150 cells per culture) of SMCs had phagocytosed ≥1 microbead, with 19% (9%) having taken up ≥5 microbeads and 2.7% (0.9%) ≥18 microbeads. Occasionally, a SMC phagocytosed very large numbers of beads (Fig. 9 *Ab*), which it clustered around the perinuclear region.

### Modulated macrophage‐like SMCs do not stain for macrophage markers or take up AcLDL but do show lower levels of SMA expression

Despite the macrophage‐like phagocytic activity, no clear staining for the classic macrophage marker CD68 was observed in any of the tracked SMCs that were stained, whether from aorta, CA, PV or colon (any fluorescence after staining for CD68 was highly diffuse and around background levels). CD68 antibody reactivity and specificity was confirmed by staining freshly isolated peritoneal cavity macrophages (supporting information for review purposes). Neither was there evidence of staining for the macrophage marker F4/80 when SMCs isolated from mouse colon were studied. Nor did SMCs take up fluorescently labelled AcLDL following phenotypic modulation (Fig. 9 *B*). In contrast, patches of ECs tracked from the fully differentiated cell type accumulated AcLDL readily (Fig. 9 *B* and Movie 9 in Supporting information; EC identification was carried out by von Willebrand factor staining, Supporting Information for review purposes).

When freshly isolated CA SMCs and SMCs that had been in culture for 1 week were stained for SMA (Fig. 9 *C*), a significant decrease (*P* < 0.05 Mann‐Whitney) in SMA expression was observed when compared to native cells (normalised to native cells, median SMA intensity was 0.19 with range 0.15–0.29). This is consistent with the literature (Campbell *et al*. 1989). Despite this decrease, cultured SMCs still showed clear SMA staining with distinct stress fibres. In comparison, tracked cells not of SM origin showed weak to no staining after several days in culture (e.g. Fig. 8 *Bc* and *d*). Interestingly SM‐MHC expression (Fig. 9 *D*) did not decrease after 1 week in culture and there was rather a small increase (*P* < 0.05 Mann‐Whitney) in fluorescence (normalised to native cells, median SM‐MHC intensity was 1.36 with range 1.19–1.52). However, with native SMCs there was a large range of SM‐MHC fluorescence levels which included SMCs with high levels of SM‐MHC expression. These high levels were not present after 1 week and the interquartile range was reduced (Fig. 9 *D*).

## Discussion

Macrophage and SMCs are widely believed to contribute to the formation of neointimal plaques in atherosclerosis. However, in atherosclerotic plaques, those cells classified as macrophage and ‘foam cells’ (lipid‐laden macrophage) may also express SMA and SM22α – markers usually associated with SM (Mietus‐Snyder *et al*. 2000; Allahverdian *et al*. 2014). The observation that macrophage‐like cells express SM markers led to the proposal that SM itself may become a macrophage (Gomez *et al*. 2013; Allahverdian *et al*. 2014; Feil *et al*. 2014; Shankman *et al*. 2015), with SM reprogramming from a contractile to a migratory cell in the process of phenotypic modulation. However, there is an absence of direct evidence for phenotypic modulation and monocyte‐derived macrophage may also express SMA and SM22α (Martin *et al*. 2009).

Rather than SM, several progenitor cell types derived from the vascular wall have also been proposed to underlie neointimal formation (Margariti *et al*. 2006). In these proposals, fully differentiated SMCs may play no role in vascular remodelling and other (progenitor) cells in the vascular wall may be rapidly induced to express SM markers, e.g. SMA (Sainz *et al*. 2006; Tang *et al*. 2012). These progenitor cells may also give rise to cultures thought to derive from SM (Tang *et al*. 2012, 2013). A difficulty in unequivocally identifying the cells underlying plaque formation, and those cells studied in culture assumed to be SMCs, is ambiguity in the markers used to identify cells. Markers associated with SM may also be found in several other cell types (Shapland *et al*. 1988; Arciniegas *et al*. 1992; Basson *et al*. 1992; Moroianu *et al*. 1993; Sartore *et al*. 2001; Martin *et al*. 2009; Ludin *et al*. 2012; Shen *et al*. 2012; Karagianni *et al*. 2013).

To address the question of whether or not a fully differentiated contractile SMC may become a macrophage‐like cell we tracked the same native SMCs continuously, in prolonged time‐lapse imaging, to determine if phenotypic modulation giving rise to different functional behaviours occurred. The results show fully differentiated SMC convert readily from contractile to migratory phenotypes. The migratory SMCs were capable of significant phagocytosis, ingesting cell fragments and fluorescent microbeads. The migratory SMCs also communicated with nearby cells via the formation of tunnelling nanotubes and extrusion of microparticles. This substantial change in phenotype and function occurred over a remarkably short time frame (at least in these standard culture conditions) and SMCs began phagocytosing extracellular material as early as ∼48 h after induction, though typically 3–4 days where required. These results unambiguously establish that SMC are capable of reprogramming to a different functional behaviour.

In the present study, freshly isolated SMCs were relaxed and had low intracellular resting [Ca^2+^]_c_. In response to agonists, [Ca^2+^]_c_ increased and contraction occurred. In standard culture conditions in the presence of serum, the process of phenotypic modulation occurred following a consistent sequence of events: the SMCs first rounded up, before extending cellular processes, spreading fully then becoming migratory. Whilst spreading, small scale contractile activity (beating) occurred in PV and colon SMCs, but not in CA or aorta. For PV and colon, this beating may provide a useful identifying feature of SMCs in mixed cell populations.

Concomitant with spreading was the loss of response to the SMC agonists PE/CCh, with a steady decline in the number of cells exhibiting a Ca^2+^ response over the first few days in culture. By day 6, no cells responded. The contractile response disappeared even more quickly and was largely lost by day 3. This suggests either a change in intracellular Ca^2+^ handling mechanisms, significant receptor loss or both. Previous studies investigating bladder and colonic SMCs have reported significant receptor loss in cultured cells (Ennes *et al*. 1992; Bahadory *et al*. 2013), as well as a decrease in InsP_3_ production (Boselli *et al*. 2002). Our results also showed a significant drop in the levels of SMA expressed after 1 week in culture, though clear SMA stress fibres were still apparent in the majority of cells. Unexpectedly, when SM‐MHC was quantified, there was no decrease in SM‐MHC staining after 1 week and a small but significant increase occurred. This may reflect the relatively slow turnover of the protein and it may be influenced by the survival of only a sub‐population of the starting native SMCs (as only around 15% of CA cells survived) which had widely varying levels of SM‐MHC expression.

Migratory SMCs showed the clear ability to phagocytose cellular fragments. To confirm that they were truly internalising extracellular material, they were provided with fluorescent beads. 3D imaging established that beads were internalised by migratory SMCs, whilst analysis of larger populations showed that the majority of SMCs demonstrated phagocytic activity and that a small percentage of cells could phagocytose large numbers of beads. This phagocytic activity displayed by the migratory SM appears similar to the functional activity of a macrophage cell. However, fibroblasts may also display phagocytic behaviour, and ingest IgG‐ or collagen‐coated microbeads (Arlein *et al*. 1998; Jiang & Grinnell, 2005) and the migratory SMCs could instead be behaving as a phagocytic fibroblast‐like cell.

Macrophages are usually thought to be derived from monocytes but are now recognised to take on several forms (e.g. microglia, Kupffer cells and osteoclasts) and macrophage replenishment may occur by local macrophage proliferation (Robbins *et al*. 2013). It is tempting to speculate that SM may have the capacity to act in a macrophage‐like role (Gomez *et al*. 2013; Allahverdian *et al*. 2014; Feil *et al*. 2014). Several lines of evidence support this proposal. Cholesterol loading of cultured SMCs was found to suppress SM markers and activate macrophage markers (Rong *et al*. 2003) by downregulating miR‐143/145 (Vengrenyuk *et al*. 2015). In lineage tracing experiments, using SM22α as a marker, medial SMCs were found to convert to macrophage‐like cells that have lost classic SMC marker expression (Feil *et al*. 2014). SMCs have also previously been reported to convert to a macrophage‐like phenotype that stained positive for macrophage markers such as CD36 and CD68 (Matsumoto *et al*. 2000) or MAC‐2 (Feil *et al*. 2004, 2014). However, unambiguous identification of the source cell type for those expressing SM and macrophage markers is problematic: macrophages (and monocytes) themselves may stain for SMα‐actin and SM22α (Ludin *et al*. 2012; Shen *et al*. 2012) and vascular non‐SMC may be induced to express SM markers (Tang *et al*. 2012), whilst there may be adventitial and medial progenitor cells giving rise to rapidly proliferating cells that express SM markers (reviewed by Wang *et al*. 2015).

In the present study, those SMCs showing phagocytic behaviour did not stain for CD68 or F4/80. Perhaps additional stimuli (e.g. cholesterol loading) are required to induce expression in our experimental conditions. It is interesting in this context that macrophage markers were not previously detected in cultured cells in the absence of cholesterol loading (Shankman *et al*. 2015). It is also noteworthy that tracked SMCs in our study showed significant phagocytic activity in the complete absence of cholesterol loading; in other studies cholesterol loading was required to induce this macrophage‐like behaviour in cells maintained in culture (Rong *et al*. 2003; Shankman *et al*. 2015; Vengrenyuk *et al*. 2015). This observation suggests that SMC could demonstrate phagocytic behaviour and macrophage‐like characteristics in the absence of conventional macrophage markers and of plaque forming stimuli like cholesterol.

The class AI/II scavenger receptors may participate in macrophage foam cell formation (Takahashi *et al*. 2002). Class AI/II scavenger receptors in SMC may also contribute the uptake of LDL and in particular AcLDL (Li *et al*. 1995). However, in the present study SMCs did not take up fluorescently labelled AcLDL following phenotypic modulation. In contrast, patches of ECs tracked from the fully differentiated cell type accumulated AcLDL readily.

When migratory, the phenotypically modulated SMCs made transient connections with other nearby cells, in the form of contacting processes or TNTs (long thin tubes of membrane forming cell‐cell connections). In other cell types, vesicles derived from various organelles (Kadiu & Gendelman, 2011 *a*,*b*; Wang *et al*. 2011), or containing plasma membrane components (Rustom *et al*. 2004), cytoplasmic molecules, Ca^2+^ (Watkins & Salter, 2005; Smith *et al*. 2011), pathogens (bacteria (Onfelt *et al*. 2004), HIV particles (Sowinski *et al*. 2008) and prions (Gousset *et al*. 2009)) and mitochondria (Koyanagi *et al*. 2005; Davis & Sowinski, 2008; Gerdes & Carvalho, 2008; Abounit & Zurzolo, 2012) have been reported as being transferred via TNTs. TNTs may also associate with gap junctions to permit electrical coupling among remote cells (Wang & Gerdes, 2012) and may constitute a route of intercellular signalling during development, immune responses and regeneration processes. Our results suggest that TNTs may also be an important form of communication for phenotypically modified SMCs.

Migratory SMCs also transferred material via microparticle‐like structures in a process that was both frequent and rapid. The microparticles may include mitochondria. Transfer of material via microparticles is also a recognised regulator of cell‐to‐cell interactions (Ratajczak *et al*. 2006 *b*) in several cell types (e.g. platelets, monocytes, ECs (Mause & Weber, 2010; Chaar *et al*. 2011)) including SM (Bobryshev *et al*. 2013) and may be a contributor to the pathogenesis of vascular disease. Indeed, microparticles derived from ECs may be diagnostic markers of EC dysfunction in vascular diseases (Boulanger, 2010) while microparticles from platelets may promote angiogenesis (Varon & Shai, 2009). Microparticles can alter gene expression in target cells by transferring mRNA and miRNA (Ratajczak *et al*. 2006 *a*). Significantly, the phenotypic development of stem cells can be controlled via microparticles (Ankrum *et al*. 2014). Microparticle transfer may contribute similarly to cell phenotype development in vascular disease.

In this study we show that SMCs have the capability to undergo significant phenotypic modulation. Contractile SMCs were shown to rapidly develop new functional capabilities, which include the ability to migrate and to phagocytose foreign material, and it is tempting to speculate that SMCs may be a potential source of macrophages in vascular remodelling.

## Additional information

### Competing interests

None declared.

### Author contributions

All authors approve the manuscript and qualify for its authorship. All experiments were performed at the Strathclyde Institute of Pharmacy and Biomedical Sciences. M.E.S. contributed to the experimental design, acquisition of the experimental data, data analysis and writing the manuscript; J.D. contributed to the development of the imaging system, data analysis and to manuscript revisions; J.McC. contributed to the experimental design, development of the imaging system, data interpretation and writing the manuscript.

### Funding

This work was funded by the Wellcome Trust (092292/Z/10/Z; 202924/Z/16/Z) and British Heart Foundation (PG/11/70/29086; PG/16/54/32230).

### Author's present address

M. E. Sandison: Department of Biomedical Engineering University of Strathclyde Wolfson Centre 106 Rottenrow Glasgow G4 0NW, UK.

## Supporting information


**Movie 1**. Agonist‐induced contraction and subsequent phenotypic modulation of a colonic SMC. Corresponding to Fig. 3A (with the same length and [Ca^2+^]_c_ scales), this movie first shows a colonic SMC contracting in response to two puffs of CCh, with a strong [Ca^2+^]_c_ rise resulting from each puff (as shown in false colour on the right). After allowing the SMC to relax, the buffer in the culture dish was replaced with serum‐containing media and the same SMC was continuously tracked as it underwent phenotypic modulation, rounding up fully then extending elongated processes outwards (starting at ∼28 h), before spreading and becoming motile. In this movie two other cells can also be seen in the FOV: a second SMC that exhibited a weak [Ca^2+^]_c_ increase in response to CCh and which spread outwards at later time‐point (extending process outwards at ∼75 h) and a small round cell that was not a SMC but which spread and became motile within the first day. After initially recording in bright‐field mode, all recordings were in phase contrast from 22 h. During a media change at ∼2 h a dead cell that had flowed into the FOV during the addition of media was washed away, whilst during a media change at ∼75 h a large cluster of cellular debris was washed into the FOV.
**Movie 2**. Phenotypic modulation of a PV SMC. Corresponding to Fig. 3*B* (with the same length scales), this movie tracks a freshly isolated PV SMC as it undergoes phenotypic modulation in culture conditions. After spreading and becoming motile, the SMC appears to phagocytose some nearby extracellular debris at ∼48 h (yellow arrow indicates debris). Another smaller cell with a morphology different to that of a SMC, which spread with the first few hours of being in culture, can also be seen in the FOV (unlike all PV SMCs tracked, this cell did not undergo a period of spontaneous contraction).
**Movie 3**. Phenotypic modulation of a CA SMC. Corresponding to Fig. 3*C* (with the same length scales), this movie tracks a freshly isolated PV SMC as it undergoes phenotypic modulation in culture conditions. Two CA SMCs can be seen in the FOV: the tracked SMC that initially begins to spread, then re‐rounds before eventually fully spreading and becoming motile; and a second SMC that undergoes apoptosis at ∼6 h.
**Movie 4**. Spontaneous contractions occurring during phenotypic modulation of PV SMCs. Corresponding to Figure 4*A–C*, this movie provides examples of the spontaneous contractions that PV SMCs exhibit during their transition to a migratory phenotype. The first section shows phase contrast recordings of four different SMCs (2 min recording burst shown for each SMC) as they contract during their transition (recorded at 45h47, 37h47, 27h47 and 31h47; the fourth cell corresponds to Fig.4*C*, green trace and *Ca*). The second section shows the fourth cell 10 h later (Fig. 4*C*, blue trace and *Cb*). The subsequent movie section shows the spontaneous [Ca^2+^]c oscillations, as visualised by Fluo‐4 fluorescence, that accompanied the onset of cell spreading and early spontaneous contractions in the SMC shown in Fig. 4 *Aa* and *b* (the traces in *Ca–c* are derived from this recording).
**Movie 5**. Tracking the migration of a colonic SMC. Corresponding to Fig. 5, this movie shows the onset of the migratory behaviour of a tracked colonic SMC. The right hand side of the first movie section shows the Histone 2B‐GFP images used for tracking and the expression of the protein can be seen to rise with the onset of motility. Despite the Histone 2B CellLights reagent having been present in the culture media from the beginning of the experiment, protein expression was only observed from ∼92 h once the cell had fully spread. As the SMC began to move around, it was observed taking up and engulfing extracellular debris, including a large fragment of debris at the bottom of the FOV. When viewed at a slower speed (second movie section), the SMC can be seen to first reel in the cell debris before undergoing a series of strong contractions during which it appears to ingest the fragment. It can also be seen that, as the cell moves around, it occasionally leaves behind subcellular fragments of its own (e.g. at around 36 s).
**Movie 6**. Contraction of PV SMCs in response to PE during phenotypic modulation. Corresponding to Fig. 7, this movie of the [Ca^2+^]c response as measured by Fluo‐4 shows the contractions exhibited by of one the two SMCs puffed with PE after 47 h in culture (corresponding to the black trace and brightfield image in Fig. 7*A*) and the SMC puffed at 119 h (Fig. 7*B*). The movies are temporally aligned so that both puffs begin ∼4 s into the movie, which runs at a speed of x8. Prior to the puff, spontaneous [Ca^2+^]c oscillations can be observed in the 47 h SMC and a clear difference in the speed of the contractions occurring on day 2 and on day 5 can be seen.
**Movie 7**. Phagocytosis of a dead cell by a recently contractile PV SMC. Corresponding to Fig. 8*A*, this movie tracks a PV SMC, whose contractility was first confirmed by the puffing of PE (with the cell in serum‐free media). The three other SMCs in the FOV can also be seen to contract. FBS was then added to the culture dish, immediately before the start of the relevant movie section, during which two more SMCs were washed into the FOV. In response to FBS, all SMCs immediately started to contract down, with a substantial loss of elongation after 10 min. The SMCs then rounded up fully before starting to spread outwards, with the six SMCs in the FOV spreading at varying times. After 25 h in culture the tracked SMC had spread outwards and was spontaneously contracting. Several smaller, round cells that are not SMCs (but are present in the SM tissue) can also be seen in the initial FOV. One of these was close to the tracked SMC and at ∼39 h it underwent apoptosis. Shortly afterwards, at ∼48 h, the SMC was clearly observed to take up and engulf this dead cell.
**Movie 8**. Phagocytosis of fluorescent microbeads by tracked SMCs. This movie shows two examples of the phagocytosis of fluorescent beads by migratory SMCs, monitored by simultaneous phase contrast/fluorescence time‐lapse imaging, the first from a tracked colon cell and the second from a tracked PV cell. In both cases the upper panel shows the phase contrast images and the lower panel shows the microbead fluorescence. The beads are discernible in phase contrast appearing as white dots but, as other structures have a similar appearance, the fluorescence signal is required to confirm which structures are beads. The colon example comes from a later time‐point in the tracking of the SMC shown in Fig. 3A and Movie 1, whose contractility was confirmed at the outset of the experiment by CCh puffing. Here, the SMC first reels in a bead at the top of the image (to the left of the centre, hovering over another cell) and internalises it (confirmed by 3D reconstruction microscopy), before taking up a second bead that lands on the righthand side of the image shortly after the start of the movie. In the PV example, the SMC can be seen to similarly phagocytose a single bead that lands on the right of the image.
**Movie 9**. Uptake of AcLDL by endothelial cells but not motile SMCs. Corresponding to Fig. 9*B*, the first section of this movie shows a patch of endothelial cells taking up AcLDL (left, brightfield; middle, fluorescently labelled AcLDL) over an 18 h period immediately following the addition of LDL to the culture. SMCs in same culture (within the same FOV) did not take up LDL, as demonstrated by the example on the right (top right, brightfield; bottom right, AcLDL). A few large fluorescent clusters can be seen to land upon the cells being imaged, including the SMC. However, the SMC did not internalise this cluster, which at the end of the recording remains in a focal plane above the cell. The second movie section consists of a high speed imaging burst that shows the intracellular trafficking of the internalised LDL.

## References

[tjp7415-bib-0001] Abounit S & Zurzolo C (2012). Wiring through tunneling nanotubes – from electrical signals to organelle transfer. J Cell Sci 125, 1089–1098. 22399801 10.1242/jcs.083279

[tjp7415-bib-0002] Allahverdian S , Chehroudi AC , McManus BM , Abraham T & Francis GA (2014). Contribution of intimal smooth muscle cells to cholesterol accumulation and macrophage‐like cells in human atherosclerosis. Circulation 129, 1551–1559. 24481950 10.1161/CIRCULATIONAHA.113.005015

[tjp7415-bib-0003] Andreeva ER , Pugach IM & Orekhov AN (1997). Subendothelial smooth muscle cells of human aorta express macrophage antigen *in situ* and *in vitro* . Atherosclerosis 135, 19–27. 9395269 10.1016/s0021-9150(97)00136-6

[tjp7415-bib-0004] Ankrum JA , Miranda OR , Ng KS , Sarkar D , Xu C & Karp JM (2014). Engineering cells with intracellular agent‐loaded microparticles to control cell phenotype. Nat Protoc 9, 233–245. 24407352 10.1038/nprot.2014.002 PMC4320648

[tjp7415-bib-0005] Arciniegas E , Sutton AB , Allen TD & Schor AM (1992). Transforming growth factor beta 1 promotes the differentiation of endothelial cells into smooth muscle‐like cells *in vitro* . J Cell Sci 103, 521–529. 1478952 10.1242/jcs.103.2.521

[tjp7415-bib-0006] Arlein WJ , Shearer JD & Caldwell MD (1998). Continuity between wound macrophage and fibroblast phenotype: analysis of wound fibroblast phagocytosis. Am J Physiol *Regul Integr Comp Physiol* 275, R1041–R1048. 9756532 10.1152/ajpregu.1998.275.4.R1041

[tjp7415-bib-0007] Bahadory F , Moore KH , Liu L & Burcher E (2013). Gene expression of muscarinic, tachykinin, and purinergic receptors in porcine bladder: comparison with cultured cells. Front Pharmacol 4, 148. 24348420 10.3389/fphar.2013.00148 PMC3842897

[tjp7415-bib-0008] Basson CT , Kocher O , Basson MD , Asis A & Madri JA (1992). Differential modulation of vascular cell integrin and extracellular matrix expression *in vitro* by TGF‐beta 1 correlates with reciprocal effects on cell migration. J Cell Physiol 153, 118–128. 1522126 10.1002/jcp.1041530116

[tjp7415-bib-0009] Bennett MR , Sinha S & Owens GK (2016). Vascular smooth muscle cells in atherosclerosis. Circ Res 118, 692–702. 26892967 10.1161/CIRCRESAHA.115.306361 PMC4762053

[tjp7415-bib-0010] Bobryshev YV , Killingsworth MC & Orekhov AN (2013). Increased shedding of microvesicles from intimal smooth muscle cells in athero‐prone areas of the human aorta: implications for understanding of the predisease stage. Pathobiology 80, 24–31. 22832241 10.1159/000339430

[tjp7415-bib-0011] Bochaton‐Piallat ML , Ropraz P , Gabbiani F & Gabbiani G (1996). Phenotypic heterogeneity of rat arterial smooth muscle cell clones. Implications for the development of experimental intimal thickening. Arterioscler Thromb Vasc Biol 16, 815–820. 8640410 10.1161/01.atv.16.6.815

[tjp7415-bib-0012] Boselli C , Govoni S , Vicini D , Lanni C , Racchi M & D'Agostino G (2002). Presence and passage dependent loss of biochemical M3 muscarinic receptor function in human detrusor cultured smooth muscle cells. J Urol 168, 2672–2676. 12442008 10.1016/S0022-5347(05)64242-5

[tjp7415-bib-0013] Boulanger CM (2010). Microparticles, vascular function and hypertension. Current Opin Nephrol Hypertens 19, 177–180. 10.1097/MNH.0b013e32833640fd 20051854

[tjp7415-bib-0014] Bradley KN , Currie S , MacMillan D , Muir TC & McCarron JG (2003). Cyclic ADP‐ribose increases Ca^2+^ removal in smooth muscle. J Cell Sci 116, 4291–4306. 12966165 10.1242/jcs.00713

[tjp7415-bib-0015] Campbell GR & Campbell JH (1990). The phenotypes of smooth muscle expressed in human atheroma. Ann N Y Acad Sci 598, 143–158. 2123377 10.1111/j.1749-6632.1990.tb42286.x

[tjp7415-bib-0016] Campbell JH & Campbell GR (2012). Smooth muscle phenotypic modulation – a personal experience. Arterioscler Thromb Vasc Biol 32, 1784–1789. 22815344 10.1161/ATVBAHA.111.243212

[tjp7415-bib-0017] Campbell JH , Kocher O , Skalli O , Gabbiani G & Campbell GR (1989). Cytodifferentiation and expression of alpha‐smooth muscle actin mRNA and protein during primary culture of aortic smooth muscle cells. Correlation with cell density and proliferative state. Arteriosclerosis 9, 633–643. 2675809 10.1161/01.atv.9.5.633

[tjp7415-bib-0018] Chaar V , Romana M , Tripette J , Broquere C , Huisse MG , Hue O , Hardy‐Dessources MD & Connes P (2011). Effect of strenuous physical exercise on circulating cell‐derived microparticles. Clin Hemorheol Microcirc 47, 15–25. 21321404 10.3233/CH-2010-1361

[tjp7415-bib-0019] Chalmers S , Saunter C , Wilson C , Coats P , Girkin JM & McCarron JG (2012). Mitochondrial motility and vascular smooth muscle proliferation. Arterioscler Thromb Vasc Biol 32, 3000–3011. 23104850 10.1161/ATVBAHA.112.255174 PMC3939657

[tjp7415-bib-0020] Clowes AW , Reidy MA & Clowes MM (1983). Kinetics of cellular proliferation after arterial injury. I. Smooth muscle growth in the absence of endothelium. Lab Invest 49, 327–333. 6887785

[tjp7415-bib-0021] Davis DM & Sowinski S (2008). Membrane nanotubes: dynamic long‐distance connections between animal cells. Nat Rev Mol Cell Biol 9, 431–436. 18431401 10.1038/nrm2399

[tjp7415-bib-0022] Ennes HS , McRoberts JA , Hyman PE & Snape WJ Jr (1992). Characterization of colonic circular smooth muscle cells in culture. Am J Physiol *Gastrointest Liver Physiol* 263, G365–G370. 1329528 10.1152/ajpgi.1992.263.3.G365

[tjp7415-bib-0023] Feil S , Fehrenbacher B , Lukowski R , Essmann F , Schulze‐Osthoff K , Schaller M & Feil R (2014). Transdifferentiation of vascular smooth muscle cells to macrophage‐like cells during atherogenesis. Circ Res 115, 662–667. 25070003 10.1161/CIRCRESAHA.115.304634

[tjp7415-bib-0024] Feil S , Hofmann F & Feil R (2004). SM22alpha modulates vascular smooth muscle cell phenotype during atherogenesis. Circ Res 94, 863–865. 15044321 10.1161/01.RES.0000126417.38728.F6

[tjp7415-bib-0025] Gerdes HH & Carvalho RN (2008). Intercellular transfer mediated by tunneling nanotubes. Curr Opin Cell Biol 20, 470–475. 18456488 10.1016/j.ceb.2008.03.005

[tjp7415-bib-0026] Glukhova MA , Kabakov AE , Frid MG , Ornatsky OI , Belkin AM , Mukhin DN , Orekhov AN , Koteliansky VE & Smirnov VN (1988). Modulation of human aorta smooth muscle cell phenotype: a study of muscle‐specific variants of vinculin, caldesmon, and actin expression. Proc Natl Acad Sci USA 85, 9542–9546. 3143999 10.1073/pnas.85.24.9542 PMC282790

[tjp7415-bib-0027] Gomez D , Shankman LS , Nguyen AT & Owens GK (2013). Detection of histone modifications at specific gene loci in single cells in histological sections. Nat Methods 10, 171–177. 23314172 10.1038/nmeth.2332 PMC3560316

[tjp7415-bib-0028] Gonzalez MC , Arribas SM , Molero F & Fernandez‐Alfonso MS (2001). Effect of removal of adventitia on vascular smooth muscle contraction and relaxation. Am J Physiol Heart Circ Physiol 280, H2876–H2881. 11356648 10.1152/ajpheart.2001.280.6.H2876

[tjp7415-bib-0029] Gousset K , Schiff E , Langevin C , Marijanovic Z , Caputo A , Browman DT , Chenouard N , de Chaumont F , Martino A , Enninga J , Olivo‐Marin JC , Mannel D & Zurzolo C (2009). Prions hijack tunnelling nanotubes for intercellular spread. Nat Cell Biol 11, 328–336. 19198598 10.1038/ncb1841

[tjp7415-bib-0030] Hao H , Ropraz P , Verin V , Camenzind E , Geinoz A , Pepper MS , Gabbiani G & Bochaton‐Piallat ML (2002). Heterogeneity of smooth muscle cell populations cultured from pig coronary artery. Arterioscler Thromb Vasc Biol 22, 1093–1099. 12117722 10.1161/01.atv.0000022407.91111.e4

[tjp7415-bib-0031] Holifield B , Helgason T , Jemelka S , Taylor A , Navran S , Allen J & Seidel C (1996). Differentiated vascular myocytes: are they involved in neointimal formation? J Clin Invest 97, 814–825. 8609239 10.1172/JCI118481 PMC507120

[tjp7415-bib-0032] Jiang H & Grinnell F (2005). Cell‐matrix entanglement and mechanical anchorage of fibroblasts in three‐dimensional collagen matrices. Mol Biol Cell 16, 5070–5076. 16107563 10.1091/mbc.E05-01-0007 PMC1266407

[tjp7415-bib-0033] Kadiu I & Gendelman HE (2011 *a*). Human immunodeficiency virus type 1 endocytic trafficking through macrophage bridging conduits facilitates spread of infection. J Neuroimmune Pharmacol 6, 658–675. 21789505 10.1007/s11481-011-9298-z PMC3232570

[tjp7415-bib-0034] Kadiu I & Gendelman HE (2011 *b*). Macrophage bridging conduit trafficking of HIV‐1 through the endoplasmic reticulum and Golgi network. J Proteome Res 10, 3225–3238. 21563830 10.1021/pr200262q PMC3128463

[tjp7415-bib-0035] Kamishima T & McCarron JG (1998). Ca^2+^ removal mechanisms in rat cerebral resistance size arteries. Biophys J 75, 1767–1773. 9746518 10.1016/S0006-3495(98)77618-0 PMC1299848

[tjp7415-bib-0036] Karagianni F , Prakoura N , Kaltsa G , Politis P , Arvaniti E , Kaltezioti V , Psarras S , Pagakis S , Katsimboulas M , Abed A , Chatziantoniou C & Charonis A (2013). Transgelin up‐regulation in obstructive nephropathy. PLoS One 8, e66887. 23840546 10.1371/journal.pone.0066887 PMC3694161

[tjp7415-bib-0037] Koyanagi M , Brandes RP , Haendeler J , Zeiher AM & Dimmeler S (2005). Cell‐to‐cell connection of endothelial progenitor cells with cardiac myocytes by nanotubes: a novel mechanism for cell fate changes? Circ Res 96, 1039–1041. 15879310 10.1161/01.RES.0000168650.23479.0c

[tjp7415-bib-0038] Leclerc G , Isner JM , Kearney M , Simons M , Safian RD , Baim DS & Weir L (1992). Evidence implicating nonmuscle myosin in restenosis. Use of *in situ* hybridization to analyze human vascular lesions obtained by directional atherectomy. Circulation 85, 543–553. 1735150 10.1161/01.cir.85.2.543

[tjp7415-bib-0039] Li H , Freeman MW & Libby P (1995). Regulation of smooth muscle cell scavenger receptor expression *in vivo* by atherogenic diets and *in vitro* by cytokines. J Clin Invest 95, 122–133. 7814605 10.1172/JCI117628 PMC295387

[tjp7415-bib-0040] Li S , Fan YS , Chow LH , Van Den Diepstraten C , van Der Veer E , Sims SM & Pickering JG (2001). Innate diversity of adult human arterial smooth muscle cells: cloning of distinct subtypes from the internal thoracic artery. Circ Res 89, 517–525. 11557739 10.1161/hh1801.097165

[tjp7415-bib-0041] Li Z , Cheng H , Lederer WJ , Froehlich J & Lakatta EG (1997). Enhanced proliferation and migration and altered cytoskeletal proteins in early passage smooth muscle cells from young and old rat aortic explants. Exp Mol Pathol 64, 1–11. 9203504 10.1006/exmp.1997.2204

[tjp7415-bib-0042] Ludin A , Itkin T , Gur‐Cohen S , Mildner A , Shezen E , Golan K , Kollet O , Kalinkovich A , Porat Z , D'Uva G , Schajnovitz A , Voronov E , Brenner DA , Apte RN , Jung S & Lapidot T (2012). Monocytes‐macrophages that express alpha‐smooth muscle actin preserve primitive hematopoietic cells in the bone marrow. Nature Immunol 13, 1072–1082. 22983360 10.1038/ni.2408

[tjp7415-bib-0043] McCarron JG & Muir TC (1999). Mitochondrial regulation of the cytosolic Ca^2+^ concentration and the InsP_3_‐sensitive Ca^2+^ store in guinea‐pig colonic smooth muscle. J Physiol 516, 149–161. 10066930 10.1111/j.1469-7793.1999.149aa.x PMC2269201

[tjp7415-bib-0044] Madsen CS , Regan CP , Hungerford JE , White SL , Manabe I & Owens GK (1998). Smooth muscle‐specific expression of the smooth muscle myosin heavy chain gene in transgenic mice requires 5′‐flanking and first intronic DNA sequence. Circ Res 82, 908–917. 9576110 10.1161/01.res.82.8.908

[tjp7415-bib-0045] Margariti A , Zeng L & Xu Q (2006). Stem cells, vascular smooth muscle cells and atherosclerosis. Histol Histopathol 21, 979–985. 16763948 10.14670/HH-21.979

[tjp7415-bib-0046] Martin K , Weiss S , Metharom P , Schmeckpeper J , Hynes B , O'Sullivan J & Caplice N (2009). Thrombin stimulates smooth muscle cell differentiation from peripheral blood mononuclear cells via protease‐activated receptor‐1, RhoA, and myocardin. Circ Res 105, 214–218. 19574550 10.1161/CIRCRESAHA.109.199984

[tjp7415-bib-0047] Matsumoto K , Hirano K , Nozaki S , Takamoto A , Nishida M , Nakagawa‐Toyama Y , Janabi MY , Ohya T , Yamashita S & Matsuzawa Y (2000). Expression of macrophage (Mphi) scavenger receptor, CD36, in cultured human aortic smooth muscle cells in association with expression of peroxisome proliferator activated receptor‐gamma, which regulates gain of Mphi‐like phenotype *in vitro*, and its implication in atherogenesis. Arterioscler Thromb Vasc Biol 20, 1027–1032. 10764668 10.1161/01.atv.20.4.1027

[tjp7415-bib-0048] Mause SF & Weber C (2010). Microparticles: protagonists of a novel communication network for intercellular information exchange. Circ Res 107, 1047–1057. 21030722 10.1161/CIRCRESAHA.110.226456

[tjp7415-bib-0049] Mietus‐Snyder M , Gowri MS & Pitas RE (2000). Class A scavenger receptor up‐regulation in smooth muscle cells by oxidized low density lipoprotein. J Biol Chem 275, 17661–17670. 10837497 10.1074/jbc.275.23.17661

[tjp7415-bib-0050] Moroianu J , Fett JW , Riordan JF & Vallee BL (1993). Actin is a surface component of calf pulmonary artery endothelial cells in culture. Proc Natl Acad Sci USA 90, 3815–3819. 8483899 10.1073/pnas.90.9.3815 PMC46396

[tjp7415-bib-0051] Nguyen AT , Gomez D , Bell RD , Campbell JH , Clowes AW , Gabbiani G , Giachelli CM , Parmacek MS , Raines EW , Rusch NJ , Speer MY , Sturek M , Thyberg J , Towler DA , Weiser‐Evans MC , Yan C , Miano JM & Owens GK (2013). Smooth muscle cell plasticity: fact or fiction? Circ Res 112, 17–22. 23093573 10.1161/CIRCRESAHA.112.281048 PMC4135725

[tjp7415-bib-0052] Olson ML , Sandison ME , Chalmers S & McCarron JG (2012). Microdomains of muscarinic acetylcholine and Ins(1,4,5)P_3_ receptors create ‘Ins(1,4,5)P_3_ junctions’ and sites of Ca^2+^ wave initiation in smooth muscle. J Cell Sci 125, 5315–5328. 22946060 10.1242/jcs.105163 PMC3561854

[tjp7415-bib-0053] Onfelt B , Nedvetzki S , Yanagi K & Davis DM (2004). Cutting edge: Membrane nanotubes connect immune cells. J Immunol 173, 1511–1513. 15265877 10.4049/jimmunol.173.3.1511

[tjp7415-bib-0054] Pauletto P , Chiavegato A , Giuriato L , Scatena M , Faggin E , Grisenti A , Sarzani R , Paci MV , Fulgeri PD & Rappelli A (1994). Hyperplastic growth of aortic smooth muscle cells in renovascular hypertensive rabbits is characterized by the expansion of an immature cell phenotype. Circ Res 74, 774–788. 8156626 10.1161/01.res.74.5.774

[tjp7415-bib-0055] Rainbow RD , Macmillan D & McCarron JG (2009). The sarcoplasmic reticulum Ca^2+^ store arrangement in vascular smooth muscle. Cell Calcium 46, 313–322. 19836074 10.1016/j.ceca.2009.09.001

[tjp7415-bib-0056] Ratajczak J , Miekus K , Kucia M , Zhang J , Reca R , Dvorak P & Ratajczak MZ (2006 *a*). Embryonic stem cell‐derived microvesicles reprogram hematopoietic progenitors: evidence for horizontal transfer of mRNA and protein delivery. Leukemia 20, 847–856. 16453000 10.1038/sj.leu.2404132

[tjp7415-bib-0057] Ratajczak J , Wysoczynski M , Hayek F , Janowska‐Wieczorek A & Ratajczak MZ (2006 *b*). Membrane‐derived microvesicles: important and underappreciated mediators of cell‐to‐cell communication. Leukemia 20, 1487–1495. 16791265 10.1038/sj.leu.2404296

[tjp7415-bib-0058] Robbins CS , Hilgendorf I , Weber GF , Theurl I , Iwamoto Y , Figueiredo JL , Gorbatov R , Sukhova GK , Gerhardt LM , Smyth D , Zavitz CC , Shikatani EA , Parsons M , van Rooijen N , Lin HY , Husain M , Libby P , Nahrendorf M , Weissleder R & Swirski FK (2013). Local proliferation dominates lesional macrophage accumulation in atherosclerosis. Nat Med 19, 1166–1172. 23933982 10.1038/nm.3258 PMC3769444

[tjp7415-bib-0059] Rong JX , Shapiro M , Trogan E & Fisher EA (2003). Transdifferentiation of mouse aortic smooth muscle cells to a macrophage‐like state after cholesterol loading. Proc Natl Acad Sci USA 100, 13531–13536. 14581613 10.1073/pnas.1735526100 PMC263848

[tjp7415-bib-0060] Ross R (1999). Atherosclerosis – an inflammatory disease. N Engl J Med 340, 115–126. 9887164 10.1056/NEJM199901143400207

[tjp7415-bib-0061] Rustom A , Saffrich R , Markovic I , Walther P & Gerdes HH (2004). Nanotubular highways for intercellular organelle transport. Science 303, 1007–1010. 14963329 10.1126/science.1093133

[tjp7415-bib-0062] Sainz J , Zen AA , Caligiuri G , Demerens C , Urbain D , Lemitre M & Lafont A (2006). Isolation of “side population” progenitor cells from healthy arteries of adult mice. Arterioscl Throm Vas 26, 281–286. 10.1161/01.ATV.0000197793.83391.91 16306431

[tjp7415-bib-0063] Sartore S , Chiavegato A , Faggin E , Franch R , Puato M , Ausoni S & Pauletto P (2001). Contribution of adventitial fibroblasts to neointima formation and vascular remodeling: from innocent bystander to active participant. Circ Res 89, 1111–1121. 11739275 10.1161/hh2401.100844

[tjp7415-bib-0064] Sartore S , Franch R , Roelofs M & Chiavegato A (1999). Molecular and cellular phenotypes and their regulation in smooth muscle. Rev Physiol Biochem Pharmacol 134, 235–320. 10087911 10.1007/3-540-64753-8_6

[tjp7415-bib-0065] Shankman LS , Gomez D , Cherepanova OA , Salmon M , Alencar GF , Haskins RM , Swiatlowska P , Newman AA , Greene ES , Straub AC , Isakson B , Randolph GJ & Owens GK (2015). KLF4‐dependent phenotypic modulation of smooth muscle cells has a key role in atherosclerotic plaque pathogenesis. Nat Med 21, 628–637. 25985364 10.1038/nm.3866 PMC4552085

[tjp7415-bib-0066] Shapland C , Lowings P & Lawson D (1988). Identification of new actin‐associated polypeptides that are modified by viral transformation and changes in cell shape. J Cell Biol 107, 153–161. 2839517 10.1083/jcb.107.1.153 PMC2115168

[tjp7415-bib-0067] Shen Z , Li C , Frieler RA , Gerasimova AS , Lee SJ , Wu J , Wang MM , Lumeng CN , Brosius FC 3rd , Duan SZ & Mortensen RM (2012). Smooth muscle protein 22 alpha‐Cre is expressed in myeloid cells in mice. Biochem Biophys Res Commun 422, 639–642. 22609406 10.1016/j.bbrc.2012.05.041 PMC3377770

[tjp7415-bib-0068] Smith IF , Shuai J & Parker I (2011). Active generation and propagation of Ca^2+^ signals within tunneling membrane nanotubes. Biophys J 100, L37–39. 21504718 10.1016/j.bpj.2011.03.007 PMC3077701

[tjp7415-bib-0069] Sowinski S , Jolly C , Berninghausen O , Purbhoo MA , Chauveau A , Kohler K , Oddos S , Eissmann P , Brodsky FM , Hopkins C , Onfelt B , Sattentau Q & Davis DM (2008). Membrane nanotubes physically connect T cells over long distances presenting a novel route for HIV‐1 transmission. Nat Cell Biol 10, 211–219. 18193035 10.1038/ncb1682

[tjp7415-bib-0070] Stemerman MB & Ross R (1972). Experimental arteriosclerosis. I. Fibrous plaque formation in primates, an electron microscope study. J Exp Med 136, 769–789. 4626850 10.1084/jem.136.4.769 PMC2139284

[tjp7415-bib-0071] Stewart HJ , Guildford AL , Lawrence‐Watt DJ & Santin M (2009). Substrate‐induced phenotypical change of monocytes/macrophages into myofibroblast‐like cells: a new insight into the mechanism of in‐stent restenosis. J Biomed Mater Res A 90, 465–471. 18546184 10.1002/jbm.a.32100

[tjp7415-bib-0072] Takahashi K , Takeya M & Sakashita N (2002). Multifunctional roles of macrophages in the development and progression of atherosclerosis in humans and experimental animals. Med Electron Microsc 35, 179–203. 12658354 10.1007/s007950200023

[tjp7415-bib-0073] Tang Z , Wang A , Wang D & Li S (2013). Smooth muscle cells: to be or not to be? Response to Nguyen *et al* . Circ Res 112, 23–26. 23093574 10.1161/CIRCRESAHA.112.281055

[tjp7415-bib-0074] Tang Z , Wang A , Yuan F , Yan Z , Liu B , Chu JS , Helms JA & Li S (2012). Differentiation of multipotent vascular stem cells contributes to vascular diseases. Nature Commun 3, 875. 22673902 10.1038/ncomms1867 PMC3538044

[tjp7415-bib-0075] Varon D & Shai E (2009). Role of platelet‐derived microparticles in angiogenesis and tumor progression. Discov Med 8, 237–241. 20040277

[tjp7415-bib-0076] Vengrenyuk Y , Nishi H , Long X , Ouimet M , Savji N , Martinez FO , Cassella CP , Moore KJ , Ramsey SA , Miano JM & Fisher EA (2015). Cholesterol loading reprograms the microRNA‐143/145‐myocardin axis to convert aortic smooth muscle cells to a dysfunctional macrophage‐like phenotype. Arterioscler Thromb Vasc Biol 35, 535–546. 25573853 10.1161/ATVBAHA.114.304029 PMC4344402

[tjp7415-bib-0077] Wang G , Jacquet L , Karamariti E & Xu Q (2015). Origin and differentiation of vascular smooth muscle cells. J Physiol 593, 3013–3030. 25952975 10.1113/JP270033 PMC4532522

[tjp7415-bib-0078] Wang X & Gerdes HH (2012). Long‐distance electrical coupling via tunneling nanotubes. Biochim Biophys Acta 1818, 2082–2086. 21930113 10.1016/j.bbamem.2011.09.002

[tjp7415-bib-0079] Wang Y , Cui J , Sun X & Zhang Y (2011). Tunneling‐nanotube development in astrocytes depends on p53 activation. Cell Death Differ 18, 732–742. 21113142 10.1038/cdd.2010.147 PMC3131904

[tjp7415-bib-0080] Watkins SC & Salter RD (2005). Functional connectivity between immune cells mediated by tunneling nanotubules. Immunity 23, 309–318. 16169503 10.1016/j.immuni.2005.08.009

