# Supplementary material for: The transition of smooth muscle cells from a contractile to a migratory, phagocytic phenotype: direct demonstration of phenotypic modulation
Source: J Physiol. 2016 Aug 13;594(21):6189–209. doi: 10.1113/JP272729 (PMC5088226; doi:10.1113/JP272729)
Supplement: Supplementary file 1 — Movie 1. Agonist‐induced contraction and subsequent phenotypic modulation of a colonic SMC. Corresponding to Fig. 3A (with the same length and [Ca2+]c scales), this movie first shows a colonic SMC contracting in response to two puffs of CCh, with a strong [Ca2+]c rise resulting from each puff (as shown in false colour on the right). After allowing the SMC to relax, the buffer in the culture dish was replaced with serum‐containing media and the same SMC was continuously tracked as it underwent phenotypic modulation, rounding up fully then extending elongated processes outwards (starting at ∼28 h), before spreading and becoming motile. In this movie two other cells can also be seen in the FOV: a second SMC that exhibited a weak [Ca2+]c increase in response to CCh and which spread outwards at later time‐point (extending process outwards at ∼75 h) and a small round cell that was not a SMC but which spread and became motile within the first day. After initially recording in bright‐field mode, all recordings were in phase contrast from 22 h. During a media change at ∼2 h a dead cell that had flowed into the FOV during the addition of media was washed away, whilst during a media change at ∼75 h a large cluster of cellular debris was washed into the FOV. Movie 2. Phenotypic modulation of a PV SMC. Corresponding to Fig. 3B (with the same length scales), this movie tracks a freshly isolated PV SMC as it undergoes phenotypic modulation in culture conditions. After spreading and becoming motile, the SMC appears to phagocytose some nearby extracellular debris at ∼48 h (yellow arrow indicates debris). Another smaller cell with a morphology different to that of a SMC, which spread with the first few hours of being in culture, can also be seen in the FOV (unlike all PV SMCs tracked, this cell did not undergo a period of spontaneous contraction). Movie 3. Phenotypic modulation of a CA SMC. Corresponding to Fig. 3C (with the same length scales), this movie tracks a freshly isolated P [file TJP-594-6189-s001.zip › tjp7415-sup-0001-text.pdf]

LEGENDS FOR SUPPLEMENTARY MOVIES

Please note that in all supplemental movies, where appropriate, the title slides displayed within the movies indicate the time period (following the addition of serum-containing culture media) and speed of the subsequent movie section. A red arrow on a still image of the first frame of the subsequent movie section is used to indicate the tracked SMC. During tracking experiments, there were intermittent short breaks in recording to allow for media changes (typically 5-15 min), repositioning of the FOV or changes to imaging conditions (typically 2-5 min), which are responsible for occasional small shifts in cell position.

Abbreviations used:

$[Ca^{2+}]_c$	Cytoplasmic calcium concentration
AcLDL	Acetylated low-density lipoprotein
CA	Carotid artery
CCh	Carbachol
FBS	Fetal bovine serum
FOV	Field of view
PE	Phenylephrine
PV	Portal vein
SM	Smooth muscle
SMC	Smooth muscle cell

Supplementary Movie 1. *Agonist-induced contraction and subsequent phenotypic modulation of a colonic SMC.* Corresponding to Figure 3A (with the same length and $[Ca^{2+}]_c$ scales), this movie first shows a colonic SMC contracting in response to two puffs of CCh, with a strong $[Ca^{2+}]_c$ rise resulting from each puff (as shown in false colour on the right). After allowing the SMC to relax, the buffer in the culture dish was replaced with serum-containing media and the same SMC was continuously tracked as it underwent phenotypic modulation, rounding up fully then extending elongated processes outwards (starting at ~28 h), before spreading and becoming motile. In this movie two other cells can also be seen in the FOV: a second SMC that exhibited a weak $[Ca^{2+}]_c$ increase in response to CCh and which spread outwards at later time-point (extending process outwards at ~75 h) and a small round cell that was not a SMC but which spread and became motile within the first day. After initially recording in bright-field mode, all recordings were in phase contrast from 22 h. During a media change at ~2 h a dead cell that had flowed into the FOV during the addition of media was washed away, whilst during a media change at ~75 h a large cluster of cellular was washed into the FOV.

Supplementary Movie 2. *Phenotypic modulation of a PV SMC.* Corresponding to Figure 3B (with the same length scales), this movie tracks a freshly isolated PV SMC as it undergoes phenotypic modulation in culture conditions. After spreading and becoming motile, the SMC appears to phagocytose some nearby extracellular debris at ~48 h (yellow arrow indicates debris). Another smaller cell with a morphology different to that of a SMC, which spread with the first few hours of being in culture, can also be seen in the FOV (unlike all PV SMCs tracked, this cell did not undergo a period of spontaneous contraction).

Supplementary Movie 3. *Phenotypic modulation of a CA SMC.* Corresponding to Figure 3C (with the same length scales), this movie tracks a freshly isolated PV SMC as it undergoes phenotypic modulation in culture conditions. Two CA SMCs can be seen in the FOV: the tracked SMC that initially begins to spread, then re-rounds before eventually fully spreading and becoming motile; and a second SMC that undergoes apoptosis at ~6 h.

Supplementary Movie 4. *Spontaneous contractions occurring during phenotypic modulation of PV SMCs.* Corresponding to Figure 4A-C, this movie provides examples of the spontaneous contractions

that PV SMCs exhibit during their transition to a migratory phenotype. The first section shows phase contrast recordings of four different SMCs (2 min recording burst shown for each SMC) as they contract during their transition (recorded at 45h47, 37h47, 27h47 and 31h47; the fourth cell corresponds to Figure 4C, green trace and *Ci*). The second section shows the fourth cell 10 h later (Figure 4C, blue trace and *Cii*). The subsequent movie section shows the spontaneous $[Ca^{2+}]_c$ oscillations, as visualised by Fluo-4 fluorescence, that accompanied the onset of cell spreading and early spontaneous contractions in the SMC shown in Figure 4 *Ai,ii* (the traces in *Ci-iii* are derived from this recording).

Supplementary Movie 5. *Tracking the migration of a colonic SMC.* Corresponding to Figure 5, this movie shows the onset of the migratory behaviour of a tracked colonic SMC. The right hand side of the first movie section shows the Histone 2B-GFP images used for tracking and the expression of the protein can be seen to rise with the onset of motility. Despite the Histone 2B CellLights® reagent having been present in the culture media from the beginning of the experiment, protein expression was only observed from ~92 h once the cell had fully spread. As the SMC began to move around, it was observed taking up and engulfing extracellular debris, including a large fragment of debris at the bottom of the FOV. When viewed at a slower speed (second movie section), the SMC can be seen to first reel in the cell debris before undergoing a series of strong contractions during which it appears to ingest the fragment. It can also be seen that, as the cell moves around, it occasionally leaves behind subcellular fragments of its own (e.g. at around 36 s).

Supplementary Movie 6. *Contraction of PV SMCs in response to PE during phenotypic modulation.* Corresponding to Figure 7, this movie of the $[Ca^{2+}]_c$ response as measured by Fluo-4 shows the contractions exhibited by of one the two SMCs puffed with PE after 47h in culture (corresponding to the black trace and bright field image in Figure 7A) and the SMC puffed at 119h (Figure 7B). The movies are temporally aligned so that both puffs begin ~4 s into the movie, which runs at a speed of x8. Prior to the puff, spontaneous $[Ca^{2+}]_c$ oscillations can be observed in the 47h SMC and a clear difference in the speed of the contractions occurring on day 2 and on day 5 can be seen.

Supplementary Movie 7. *Phagocytosis of a dead cell by a recently contractile PV SMC.* Corresponding to Figure 8A, this movie tracks a PV SMC, whose contractility was first confirmed by the puffing of PE (with the cell in serum-free media). The three other SMCs in the FOV can also be seen to contract. FBS was then added to the culture dish, immediately before the start of the relevant movie section, during which two more SMCs were washed into the FOV. In response to FBS, all SMCs immediately started to contract down, with a substantial loss of elongation after 10 min. The SMCs then rounded up fully before starting to spread outwards, with the six SMCs in the FOV spreading at varying times. After 25 h in culture the tracked SMC had spread outwards and was spontaneously contracting. Several smaller, round cells that are not SMCs (but are present in the SM tissue) can also be seen in the initial FOV. One of these was close to the tracked SMC and at ~39 h it underwent apoptosis. Shortly afterwards, at ~48 h, the SMC was clearly observed to take up and engulf this dead cell.

Supplementary Movie 8. *Phagocytosis of fluorescent microbeads by tracked SMCs.* This movie shows two examples of the phagocytosis of fluorescent beads by migratory SMCs, monitored by simultaneous phase contrast/fluorescence time-lapse imaging, the first from a tracked colon cell and the second from a tracked PV cell. In both cases the upper panel shows the phase contrast images and the lower panel shows the microbead fluorescence. The beads are discernible in phase contrast appearing as white dots but, as other structures have a similar appearance, the fluorescence signal is required to confirm which structures are beads. The colon example comes from a later time-point in the tracking of the SMC shown in Figure 3A and Supplemental Movie 1, whose contractility was confirmed at the outset of the experiment by CCh puffing. Here, the SMC first reels in a bead at the top of the image (to the left of the centre, hovering over another cell) and internalises it (confirmed by 3D reconstruction microscopy), before taking up a second bead that lands on the right hand side of the image shortly after the start of the movie. In the PV example, the SMC can be seen to similarly phagocytose a single bead that lands on the right of the image.

Supplementary Movie 9. *Uptake of AcLDL by endothelial cells but not motile SMCs.* Corresponding to Figure 9B, the first section of this movie shows a patch of endothelial cells taking up AcLDL (left, bright-field; middle, fluorescently-labelled AcLDL) over an 18 h period immediately following the addition of LDL to the culture. SMCs in same culture (within the same FOV) did not take up LDL, as demonstrated by the example on the right (top right, bright-field; bottom right, AcLDL). A few large fluorescent clusters can be seen to land upon the cells being imaged, including the SMC. However, the SMC did not internalise this cluster, which at the end of the recording remains in a focal plane above the cell. The second movie section consists of a high speed imaging burst that shows the intracellular trafficking of the internalised LDL.
